# Mortality correlates with tree functional traits across a wood density gradient in the Central Amazon

**DOI:** 10.3389/fpls.2025.1572767

**Published:** 2025-10-15

**Authors:** Valdiek da Silva Menezes, Bruno O. Gimenez, Cynthia L. Wright, Niro Higuchi, Claudete C. Nascimento, Fernanda B. Barros, Gustavo C. Spanner, Jardel R. Rodrigues, Nate McDowell, Adam D. Collins, Robinson I. Negrón-Juárez, Jeffrey Q. Chambers, Brent D. Newman, Adriano José Nogueira Lima, Jeffrey M. Warren

**Affiliations:** ^1^ Laboratório de Manejo Florestal (LMF), Instituto Nacional de Pesquisas da Amazônia (INPA), Manaus, Brazil; ^2^ Department of Geography, University of California, Berkeley, Berkeley, CA, United States; ^3^ Environmental Sciences Division and Climate Change Science Institute, Oak Ridge National Laboratory, Oak Ridge, TN, United States; ^4^ Southern Research Station, U.S. Department of Agriculture (USDA) Forest Service, Knoxville, TN, United States; ^5^ Laboratório de Engenharia e Artefatos da Madeira, Instituto Nacional de Pesquisas da Amazônia (INPA), Manaus, Brazil; ^6^ Atmospheric Sciences and Global Change Division, Pacific Northwest National Laboratory, Richland, WA, United States; ^7^ School of Biological Sciences, Washington State University, Pullman, WA, United States; ^8^ Earth and Environmental Sciences Division, Los Alamos National Laboratory, Los Alamos, NM, United States; ^9^ Earth and Environmental Science Area, Lawrence Berkeley National Laboratory, Berkeley, CA, United States

**Keywords:** demographics, hydraulic traits, leaf traits, plant economic spectrum, tree structure, tropical forest, wood anatomy

## Abstract

**Introduction:**

Understanding the mechanisms of tree mortality in tropical ecosystems remains challenging, in part due to the high diversity of tree species and the inherently stochastic nature of mortality. Plant functional traits offer a mechanistic link between plant physiology and performance, yet their ability to predict growth and mortality remains poorly understood. Given recent increases in tree mortality rates in the Amazon forest following extreme drought and wind events, we tested if lower wood density and acquisitive plant functional traits were associated with increased growth and mortality for common co-occurring trees in the Central Amazon.

**Methods:**

Seventeen trees of different species with similar sizes but a range in wood density (WD) and wood traits were felled, then assessed for 27 different individual functional parameters, including whole tree architecture, stem xylem anatomical and hydraulic traits and leaf traits. Traits of the individual trees were related to stand-level growth and mortality rates collected periodically over 30 years from nearby permanent inventory plots.

**Results:**

Higher wood density was associated with smaller leaf size, lower foliar base cations, lower stem water content and sapwood fraction, in agreement with the fast-slow plant economics spectrum. Lower wood density was associated with more acquisitive characteristics with greater hydraulic capacity and foliar nutrient concentrations, correlating with greater growth and mortality rates.

**Discussion:**

Our results show that lower wood density is part of a coordinated suite of traits linked to high resource acquisition, fast growth, and increased mortality risk, providing a functional framework for predicting species performance and forest vulnerability under future climate stress.

## 1 Introduction

The Amazon rainforest is the largest continuous tropical forest in the world. It contains a highly diverse tree species assemblage with large carbon storage that exerts a strong influence on global biogeochemical cycles (Higuchi et al., 2004; ter Steege et al., 2013; Flores et al., 2024). One hypothesis that explains the coexistence of such a high diversity of tree species is their adaptation to niches across a wide range of resource availability (e.g., light, temperature, water table depth, wind speed and soil nutrients) (Quesada et al., 2011; Schietti et al., 2014; Sterck et al., 2011; Gorgens et al., 2021; Sousa et al., 2022) through shifts in trait performance along different ecological groups (e.g., fast-slow growth; wood density; biomass stock) (Sullivan et al., 2025; Saatchi et al., 2007). Earth system models (ESMs) account for species diversity by grouping plants with similar structural and functional properties into plant functional types (PFTs) (Lamour et al., 2023). Functional traits, on the other hand, are defined as ‘morpho-physio-phenological traits which affect plant growth, survival and reproduction (Violle et al., 2007). In this context the study of whole tree architectural characteristics along with species-specific stem and leaf functional traits, particularly among species differing in wood density (WD), growth rates, and mortality, can better represent trade-offs that equalize competitive performance among species in ESMs (Koven et al., 2020).

Fast-growing species are characterized by low WD and high mortality rates compared to slow-growing species (King et al., 2006; Osunkoya et al., 2007; Chave et al., 2009; Brienen et al., 2020; Aguirre-Gutiérrez et al., 2025). Fast-growing species have an acquisitive trait that allows an efficient use of readily available resources, and as such they proliferate after natural or anthropogenic disturbances, such as logging, deforestation, gaps, windthrow and hurricane events (Gaui et al., 2019; Marra et al., 2014; Simonetti et al., 2023; Vargas et al., 2025). In contrast, the slow-growing species tend to present higher wood densities and are more conservative, with a tendency to conserve and store resources and nutrients, and with more investment in defense mechanisms and structural integrity leading to increased longevity (e.g., using non-structural carbohydrates (NSC) (Signori-Müller et al., 2022). Growth rate usually controls the size, age and allocation of NSC pool to storage and respiration, with faster-growing trees respiring more and storing less of their NSC (Trumbore et al., 2015), ultimately affecting carbon residence time (Asao et al., 2025). In terms of hydraulic architecture, Hajek et al., 2014 observed that increased growth rate was associated with increased branch hydraulic efficiency, but not with resistance to cavitation.

In this context of functional trait variation, leaves provide a critical interface between the plant and the atmosphere, regulating ecosystem carbon and water exchange. Although there is great diversity in leaf form, it is possible to observe a spectrum of carbon and nutrient investment responses that are associated with key functions (the leaf economic spectrum; Wright et al., 2004). In the Amazon, fast-growing species tend to have short-lived leaves and higher deciduousness (Garcia et al., 2025) as well as a large fraction of nitrogen invested in the carbon-fixing Rubisco enzyme, leading to greater photosynthetic capacity (Lamour et al., 2023). In contrast, slow-growing species tend to have relatively long-lived leaves, lower maximum photosynthetic rates, and slower growth rates (Sterck et al., 2006; Lamour et al., 2023). Even so, linking carbon uptake and growth rates solely with leaf age categories in high diversity tree communities is not recommended due to the large variation in this trait (Menezes et al., 2021). Leaves also contribute a dynamic and large fraction of the total hydraulic resistance within the soil-to-leaf continuum (Wolfe et al., 2023). They are also one of the most vulnerable organs to drought-induced cavitation (e.g., Johnson et al., 2016). In this sense, leaf traits represent a critical component in the study of tree hydraulic functioning. However, recent findings suggest that some functional traits may be limited in their ability to predict tree growth variability across environmental gradients (Rosas et al., 2021) and severe droughts (Smith-Martin et al., 2023).

In addition to leaf traits, there is variation in plant vascular system traits that link leaves to soil and regulate soil water access, root water uptake, xylem transport and transpiration (Tyree and Ewers, 1991). Diversity in tree hydraulics and architecture spans functional traits that propagate from tissue to tree scales (Sperry et al., 2008). Architectural structure is an important determinant of height extension, light capture, and mechanical stability of trees (Poorter et al., 2006). Abiotic stressors such as drought or wind, and competition with neighboring trees necessitate a balance in architectural structure and hydraulic traits along the soil-plant-atmosphere pathway to maintain light capture, efficient water movement, structural and hydraulic safety. In the tropics, a recent study found that while hydraulic architecture explains species’ soil moisture dependency, it does not directly predict mortality rates (Pivovaroff et al., 2021). Key hydraulic traits include sapwood depth, xylem vessel diameter and wood density that can vary, for example, with tree height and topographic position (Cosme et al., 2017; Hajek et al., 2014). In the Central Amazon, species from upland plateau areas are generally more adaptable to drought conditions, and, therefore, invest in a more resistant hydraulic system with higher wood density, lower mean vessel hydraulic diameter, lower mean vessel area, and smaller stem cross‐sectional sapwood area than species from wetter valley areas (Cosme et al., 2017). Functional traits of plateau species are often associated with isohydric responses that help maintain leaf water potential above critical thresholds, including deep rooting, deciduousness, and stomatal regulation (Fisher et al., 2006; Chitra-Tarak et al., 2021; Oliveira et al., 2021; Gimenez et al., 2024). In the hyper-diverse Amazon forest, variation in wood hydraulic traits, often assessed through proxies such as WD, can offer insights into species’ water-use responses across environmental gradients and drought events.

Wood density has been linked to trade-offs between growth and survival among woody plants (Kitajima and Poorter, 2008; Poorter et al., 2010), where species with low WD are generally associated with faster growth and higher hydraulic conductivity, but also greater risk of hydraulic failure (Tavares et al., 2023). In contrast, species with high WD has been related to traits that confer structural and hydraulic safety (e.g., smaller vessel diameters, slow growth, lower sapwood-specific conductivity), potentially supporting greater survival under stress (Esquivel-Muelbert et al., 2020; Gray et al., 2019; Putz et al., 1983). However, it remains unclear to what extent these anatomical traits directly influence species performance and hydraulic traits under natural conditions, with stomata density and vessel element length showing promising correlations (Simioni et al., 2023). The functional role of stem hydraulics in long-term survival is still an open question, since older trees, such as those found in the Amazon with ages reaching up to 1,400 years (Chambers et al., 1998), often lose conductivity in older xylem, suggesting that other traits or compensatory mechanisms also play critical roles (e.g., leaf or root adjustments, redundancy in conductive tissues). The evolution of hydraulic systems in trees reflects a complex balance of multiple functions such as supporting vertical growth for competitive advantage and reproductive success, while also enabling efficient water transport and minimizing the risk of hydraulic failure (e.g., xylem cavitation) (Brum et al., 2023). Mechanisms that reduce cavitation risk include osmotic regulation, stomatal control, leaf shedding, adjustments in leaf-to-xylem area ratios, and the development of smaller vessel diameters to enhance embolism resistance (Brodribb, 2009; Tng et al., 2018); However, the relationship between vessel diameter and embolism vulnerability is not explicit, as additional structural traits such as cell wall thickness and pit membrane characteristics are also important (Lens et al., 2022). Hydraulic traits vary not only among different woody organs (roots, trunk and branches, e.g., Johnson et al., 2016), but also axially within a tree, with trunk height influencing vulnerability to embolism (Domec et al., 2008; Rowland et al., 2015) and maximum hydraulic conductivity (Woodruff et al., 2008).

Wood density is directly related to mechanical, physical and anatomical characteristics (Chave et al., 2009). The base of the tree may have lower (e.g., Deng et al., 2014) or higher (e.g., Dória et al., 2019; Quilhó and Pereira, 2001; Weber and Montes, 2005) wood density or mechanical strength compared to the upper stem, dependent on traits, ontogeny or environmental factors such as wind. Thus, trees classified as lower density (<0.5 g cm^-3^) have relatively low mechanical resistance (e.g., baobab; Chapotin et al., 2006) and can have higher mortality rates due to wind events (Esteban et al., 2021; Fontes et al., 2018; Negrón-Juárez et al., 2018). For these individuals, increased mechanical resistance, *i.e.*, as WD near the crown, is thought to help withstand high wind speeds, and may reflect tension wood (Zink-Sharp, 2004; Ribeiro et al., 2016). In some ecosystems, such as the archipelago of Puerto Rico, tree species with short stature and dense wood are better adapted to frequent hurricane disturbances (Vargas et al., 2025). This condition is often found in drier environments, where the prevalence of high WD and low stature may confer mechanical stability during extreme wind events (Van Bloem et al., 2007; Helmer et al., 2023; Vargas et al., 2025), especially in topographically exposed areas (Ibanez et al., 2024).

Beyond mechanical support, vertical variation of WD may reflect chemical and anatomical changes in the wood, such as differences in cell wall structure (Ziemińska et al., 2013; Nascimento et al., 2025). Axial changes in WD may also reflect a shift in water (Goldstein et al., 1998), nutrient (Lira-Martins et al., 2022) or non-structural carbohydrate storage (e.g., Woodruff and Meinzer, 2011) that may interact to buffer daily water stress. A study with Asian tropical species concluded that hydraulic conductivity predicted growth rates better than WD (Fan et al., 2012), adding the necessity of new studies that integrate historical growth rates, hydraulics and WD. At a broader scale, wood density and hydraulic traits influence species’ growth response to drought (Serra-Maluquer et al., 2022). In South America, WD varies significantly between regions, with the East-Central Amazon where our study was conducted showing higher wood densities on average (Sullivan et al., 2025). Sullivan et al. (2025) also observed that mean annual temperature, cloud frequency and maximum cumulative water deficit were key drivers of spatial patterns in wood density. In this sense, understanding how wood density and hydraulic traits relate to mortality and growth rates is essential for a more comprehensive understanding of plant community dynamics under climate change.

Within each tree there is a balance between traits that maintain function and safety concurrently. Hydraulic traits such as P50 (xylem water potential at 50% loss of conductivity) or the hydraulic safety margin (how close plants operate to P50) are widely used to evaluate plant drought vulnerability and response (Choat et al., 2012; Tavares et al., 2023). Predominantly, research in the Amazon forest has focused on how these hydraulic thresholds vary with topographical gradients (e.g., soil texture and water table depth; Cosme et al., 2017; Fontes et al., 2020; Costa et al., 2023) or canopy position (Gimenez et al., 2019; Garcia et al., 2021). The interaction of tree functional traits with prevailing environmental conditions are reflected by varying photosynthesis, respiration and transpiration rates at local scales, thereby affecting species competition, distribution and net primary productivity (Malhi et al., 2011; Tavares et al., 2023). Interestingly, hydraulic traits specifically determine how species and individuals may respond to future climate changes (Bonal et al., 2016; McDowell et al., 2018), including longer or more acute droughts such as during El Niño events (e.g., Jiménez-Muñoz et al., 2016; Powers et al., 2020). As drought-induced mortality is increasing in tropical forests, including the Central Amazon (McDowell et al., 2018; Tavares et al., 2023), it is important to quantify the key hydraulic traits and their relationships with other tree functional traits as well as with factors such as NSC reserves (Asao et al., 2025), soil nutrient availability (Soong et al., 2020) and nutrient storage in plant tissues (e.g., wood and leaves) (Bauters et al., 2022). These insights are critical for accurately modeling forest metabolism and growth under future climate scenarios. In this context, hydraulic traits may also be linked to shifts in species distributions over time (Christoffersen et al., 2017; Lavorel and Garnier, 2002).

As such, the primary objective of this study was to test if growth and mortality in co-occurring trees in an old-growth Amazonian upland forest were related to species-specific functional traits (including wood density, hydraulic traits (e.g., xylem cell diameter, sapwood to basal area fraction), anatomical traits (e.g., % fiber content), leaf traits (e.g., leaf size, nutrient content) and other characteristics (e.g., tree architecture). We hypothesized that trees with more acquisitive traits and lower wood density would have higher growth rates and higher mortality than trees with more conservative traits and higher wood density. We harvested 17 trees of different species to explore trait-trait relationships and trait variation in context of long-term demographics of relative plant success using a 30-year record of tree growth and mortality from nearby permanent inventory plots. Results were expected to provide additional insight into tree functional traits that may be important for resilience to future climate change.

## 2 Materials and methods

### 2.1 Study site

This study was conducted in a mature forest stand within the National Institute of Amazonian Research (INPA) ZF - 2 Experimental Station (60˚ 9’ 10.17” W, 2˚ 38’ 6.28” S), northwest of Manaus, Brazil in the Central Amazon. There is a mosaic pattern in topography in the area, with sandy lowlands close to the water table and clay uplands well above the water table. Research was conducted in an upland plateau, which contained a wide diversity of trees, 70% with a diameter at breast height, (~1.3 m; DBH) between 10 and 23 cm (for all trees >10 cm; Oliveira et al., 2008). The annual precipitation averages ~2,500 mm, with the driest months of the year often occurring between July and September (Souza et al., 2021; Kunert et al., 2017). The area is covered by a dense forest canopy. Population level growth and mortality data were available from long-term monitoring plots of the BIONTE Project, which was established in 1986 (Higuchi et al., 1985; Gaui et al., 2019). For this study, trees were felled up to 500 m outside the BIONTE permanent plots.

### 2.2 Tree selection

As the Central Amazon is hyper-diverse, it is difficult to find suitable replicates for some species. As such, and to maximize the range of wood density (WD) representation, we selected individuals across many species. This increased the diversity of functional traits (e.g., wood density and xylem anatomy, which are conserved by species, e.g., Hacke and Sperry, 2001; Chave et al., 2009) represented in our study, and provided a better representation of the forest functional trait composition. By using many species instead of a few species with replicates, we were also able to select co-occurring individuals of the same size, growing in the same area and that developed under the same edaphic and environmental conditions. This helped to limit trait variation due to different growing conditions at different positions on the landscape. We focused specifically on a well-drained upland plateau area with high clay content, away from slopes and valleys where soil texture and microclimate changes. We selected 17 canopy species (n=1) between 20 and 30 cm DBH, representing the major size class of this forest, following a systematic randomized sampling at the individual level, i.e., without reference to species, but stratified across a wood density (WD) gradient (**Table 1**). To further explore the data, we present results using WD as both a gradient and as a category (low, intermediate and high). The wood density classes were representative of the range of local variation including low (0.30 – 0.50 g cm^-3^), intermediate (0.50 – 0.70 g cm^-3^) and high wood densities (0.70 – 0.90 g cm^-3^) (Melo et al., 1990). For each individual we measured a wide range of functional traits related to whole tree architectural characteristics, and stem and leaf traits as listed in **Table 2**.

**Table 1:** Wood density and size characteristics of the individual harvested study trees of 17 different species with no replicates.

Wood density Class	WD (g cm^-^³)	DBH (cm)	Height (m)	Family	Species	Growth (cm year^-1^)	Mortality (% year^-1^)
*Low wood density*	0.35	19.7	23.1	Simaroubaceae	*Simarouba amara* Aubl.	1.21	1.67
	0.37	26.5	20.2	Bignoniaceae	*Jacaranda copaia* A. Gentry	0.13	0.71
	0.42	28.0	16.2	Myristicaceae	*Virola pavonis* (A.DC.) A.C.Sm	0.15	1.37
	0.43	19.1	20.5	Malvaceae	*Sterculia excelsa* Mart.	0.20	0.67
	0.45	25.0	18.1	Urticaceae	*Pourouma myrmecophila* Ducke	0.58	2.54
*Intermediate wood density*	0.51	19.4	21.9	Fabaceae	*Tachigali paniculata* Aubl.	0.61	1.42
	0.53	28.1	28.5	Euphorbiaceae	*Hevea guianensis* Aubl.	2.21	0.61
	0.56	25.4	24.0	Malvaceae	*Scleronema micranthum* (Ducke) Ducke	0.27	0.86
	0.61	26.7	23.5	Lauraceae	*Licaria martiniana* (Mez) Kosterm.	0.07	1.40
	0.63	24.9	27.0	Fabaceae	*Inga paraensis* Ducke	0.42	1.28
	0.68	26.1	17.4	Sapotaceae	*Micropholis guyanensis* (A. DC.) PIERRE	0.14	0.89
	0.69	22.0	25.9	Annonaceae	*Guatteria olivacea* R.E.Fr.	0.54	2.05
*High wood density*	0.73	26.6	19.4	Goupiaceae	*Goupia glabra* Aubl.	0.33	0
	0.82	28.4	17.5	Olacaceae	*Minquartia guianensis* Aubl.	0.06	0.21
	0.82	21.5	21.3	Lecythidaceae	*Eschweilera coriacea* (DC.) S.A.Mori	0.13	0.25
	0.84	29.2	22.5	Lecythidaceae	*Lecythis pisonis* Cambess.	0.14	0
	0.87	28.5	21.4	Sapotaceae	*Pouteria venosa* (Mart.) Baehni	0.17	0.35

Growth and mortality rates for the same species were based on population demographics collected at adjacent long-term inventory plots.

**Table 2:** Description of whole tree architectural characteristics and stem and leaf functional traits, including code abbreviations and units.

Abbreviation	Trait	Measure position	Units
DBH	Diameter at breast height	Tree	cm
Ht	Total height	Tree	m
Sl	Stem length	Tree	m
Cl	Crown length	Tree	m
Cd	Crown diameter	Tree	m
Ce	Crown exposure	Tree	–
SWA	Sapwood area	Stem (DBH)	cm²
SWF	Sapwood to basal area fraction	Stem (DBH)	%
SWD	Sapwood depth	Stem (DBH)	cm
WD	Wood density	Stem (DBH)	g cm^-3^
WC	Wood water content	Stem (DBH)	%
Vd	Vessel diameter	Stem (DBH)	µm
VD	Vessel density	Stem (DBH)	vessel mm^-2^
VG	Vessel grouping	Stem (DBH)	–
Dh	Hydraulic diameter	Stem (DBH)	µm
Kp	Potential hydraulic conductivity	Stem (DBH)	kg m^-1^ s^-1^ MPa^-1^
Vp	Vessel area in cross section	Stem (DBH)	%
VI	Hydraulic vulnerability index	Stem (DBH)	µm mm^-2^
Pp	Parenchyma area in cross section	Stem (DBH)	%
Fp	Fiber area in cross section	Stem (DBH)	%
LS	Leaf size	Leaf	cm²
SLA	Specific leaf area	Leaf	cm² g^-1^
C	Carbon content	Leaf	%
N	Nitrogen content	Leaf	g kg^-1^
P	Phosphorus content	Leaf	g kg^-1^
K	Potassium content	Leaf	g kg^-1^
Ca	Calcium content	Leaf	g kg^-1^
Mg	Magnesium content	Leaf	g kg^-1^
N:P	Nitrogen to Phosphorus ratio	Leaf	–

### 2.3 Whole tree architecture

We measured tree diameter (DBH) at 1.3 m using a diameter tape. Crown diameter (Cd) was estimated by laying out a measurement tape along the forest floor (north-south and east-west axes) and observing vertically where the canopy edge ended. The four measurements per individual were used to estimate average Cd. Canopy exposure was estimated from the forest floor based on crown exposure to direct solar radiation based on Dawkins and Field (1978), where: 1) canopy receives little direct light; 2) canopy receives direct lateral light; 3) canopy receives overhead light on 10 – 90% of the crown; 4) canopy receives full overhead light on > 90% of the crown; 5) canopy is fully exposed with an emergent crown. The trees were felled in the early morning (7:00 - 8:30 AM) when tree water stress was minimal (to limit impacts on active sapwood area assessments, see below). Tree height (Ht) and crown length (Cl) were assessed using a measuring tape, then stem and leaf functional traits were collected and measured.

### 2.4 Stem wood hydraulic traits

#### 2.4.1 Sapwood area

After the trees were felled, we collected 40 cm long stem sub-sections (i.e., logs) for further analysis (see **Supplementary Figure 1**). To evaluate if stem traits varied along the length of the main trunk, we collected logs at three positions: one near the DBH, and the other two at 50% and 100% of the stem length (up to the base of the canopy). Sapwood area was estimated using 0.05% acid fuchsin dye dissolved in water to stain the secondary cell walls in the xylem (Umebayashi et al., 2007). Each log was placed vertically in containers with the dye solution covering ~ 5 cm of the log bottom. Over the course of the day the upper surface of the logs were exposed to evaporation, thereby pulling the dye solution into the active sapwood. Next, the bottom 10 cm of each log was removed and the average xylem radius (between inner bark and the pith) and average dyed sapwood radius measured. From these radii, the areas of the concentric circles were calculated, such that the unstained area was subtracted from the total area resulting in the active conductive sapwood area (SWA) of that section (Aparecido et al., 2019). In addition, the sapwood to basal area fraction (SWF) and sapwood depth (SWD; smallest radius) of each section were also measured (**Supplementary Figure 1**). We note that cutting may have introduced some embolism in larger vessels, but that dye was still visible in some vessels throughout the xylem, reflecting sapwood. We also note that xylem activity (as sap flow) declines quickly with depth (e.g., Spanner et al., 2022). As such, our sapwood depth measurements should be considered in that context.

#### 2.4.2 Wood density and stem water content

Wood density (WD) and saturated stem water content (WC) were measured for each tree at the Laboratório de Engenharia e Artefatos da Madeira, at INPA. A wood disc (cookie) was cut from each log, then six wood subsamples were cut from the disc: three from the 0 – 2 cm depth and three from the 6 – 8 cm depth in the radial direction. The wood samples were submerged in water for 20 days to obtain total volume and saturated mass. Subsequently, the wood samples were oven-dried at 105°C to constant dry mass. WD and WC were calculated as:


$$WD\;(g\;cm^{-3})=\frac{Dry\;mass}{Saturated\;volume}$$

$$WC\;(\%)=100*\frac{Saturated\;mass-Dry\;mass}{Dry\;mass}$$

#### 2.4.3 Wood anatomical traits

For anatomical analysis, we removed three additional wood samples from the DBH disc at the 0 – 2 cm depth interval. Histological sections were obtained using a sliding microtome (American Optical 860) and type C knives, with thickness ranging from 18 to 22 μm. Histological sections were bleached with sodium hypochlorite (20%), dehydrated in an ethanol dilution series (30%, 50%, 70% and 100%), stained with safranin, dehydrated again in an ethanol series (50%, 70% and 100%) and placed in butyl acetate for five minutes, then the slides were mounted using Entellan resin. Sections were imaged using a microscope at 40x magnification. Anatomical traits were measured using publicly available software (ImageJ v. 1.54; Rasband, 2004) and included vessel diameter (Vd; μm), vessel density (VD; number of vessels/area) and vessel grouping index (VG; total vessel number/total vessel groupings number), which is a dimensionless measure of the proportion of vessels clustered in a cross section of a tree trunk. Xylem cell types (% fibers, Fp; % parenchyma, Pp; % vessels, Vp) were measured using supervised automatic classification software (MultiSpec v. 3.5; Biehl, 2020). For all anatomical traits we averaged the mean values from the three replicate wood samples (n=3). Several example microscopy images are included in **Supplementary Figure 2**.

#### 2.4.4 Potential hydraulic conductivity

In order to describe tree water transport, we calculated the potential maximum hydraulic conductivity, *Kp* (kg m^-1^ s^-1^ MPa^-1^) and the vulnerability index, VI (µm mm-2) based on wood anatomical data (Scholz et al., 2013). *Kp* was calculated according to the Hagen-Poiseuille law (Sterck et al., 2008; Poorter et al., 2010).


$$Kp=(\frac{\pi *\rho }{128\;\eta })*VD*D_{h}^{4}$$

where, ρ is the density of water at 20°C (998.2 kg m^-3^); η is the water viscosity index at 20°C (1.002 x 10–^3^ MPa s); and the mean hydraulic diameter, Dh (μm), is calculated as:


$$D_{h}={\left[\frac{\sum Vd^{4}}{n}\right]}^{1/4}$$

where, n = number of vessels. *Kp* is intended to show how easily water can be conducted through a set of non-interacting perfectly circular conductive elements.

The vulnerability index was calculated using vessel density (vessel mm-2) and average vessel diameter (μm) (Rodríguez-Ramírez et al., 2022):


$$VI=\frac{Vd}{VD}$$

Where VI is the vulnerability index, Vd is the average vessel diameter and VD is the vessel density. Here, values near zero indicate plants are more resistant to the effects of drought.

### 2.5 Leaf traits

#### 2.5.1 Specific leaf area

To obtain specific leaf area (SLA, cm^2^ g^-1^), 30 sun leaves were collected from the upper canopy of each felled tree and measured using a portable scanner (CI - 202 Portable laser leaf area meter; CID Bio-Science). Subsequently, leaves were oven-dried at 65 °C until constant weight and ground for chemical analysis.

#### 2.5.2 Leaf chemical analysis

To assess potential trait-trait linkages between stem hydraulic anatomy and foliar physiological function we measured key foliar chemical concentrations. Foliar carbon content (C, g kg^-1^) was determined using a plasma emission spectrometer and elemental analyzer. Nitrogen (N, g kg^-1^) was determined by the Kjeldahl method. Phosphorus (P, g kg^-1^) was obtained by colorimetry and the absorbance readings performed at 660 nm using ammonium molybdate and 3% ascorbic acid. Potassium (K, g kg^-1^) was determined by flame photometry, and calcium (Ca, g kg^-1^) and magnesium (Mg, g kg^-1^) by atomic absorption spectrophotometry.

### 2.6 Growth and mortality rate

Growth and mortality rates were obtained from 12 permanent, 1-ha forest inventory plots, known as the BIONTE Project, managed by INPA (Higuchi et al., 1985). This project provided a large dataset of annual growth and mortality rates for all trees >10 cm DBH (~1500 trees). In every monitored year, additional trees were added to the inventory (recruitment), after attaining the minimum DBH of 10 cm, and the trees that died (mortality) were removed. Using this 30-year (1990 - 2020) dataset, we calculated annual diameter growth and mortality rates for our 17 selected species. We then assessed correlations between measured wood/leaf traits of our specific harvested trees and population level growth and mortality rates. As there were no replicates of tree species used in this study, conclusions about growth or mortality rates linked to traits of individual species cannot be made. Even so, regression across all 17 individuals or comparisons by density class can yield insight into trait linkages to demographics for discussion.

### 2.7 Statistical analysis

A principal component analysis (PCA) was conducted to assess the relationships between the different tree characteristics and functional traits. A correlation matrix was used to measure the degree of correlation between wood density and the functional traits. To verify if there were differences in wood density, water content, sapwood area, and sapwood fraction along the stem, an ANOVA was used. Pearson correlation was used to explore how individual functional traits may be related to long-term growth and mortality rates based on their correlation coefficients (r) and significance level (p < 0.05). All statistical analyses were performed using R 4.2.2 with the FactoMineR package used for PCA analysis (R Core Team, 2022).

## 3 Results

### 3.1 Wood density and tree, stem and leaf functional traits

**Figure 1:**
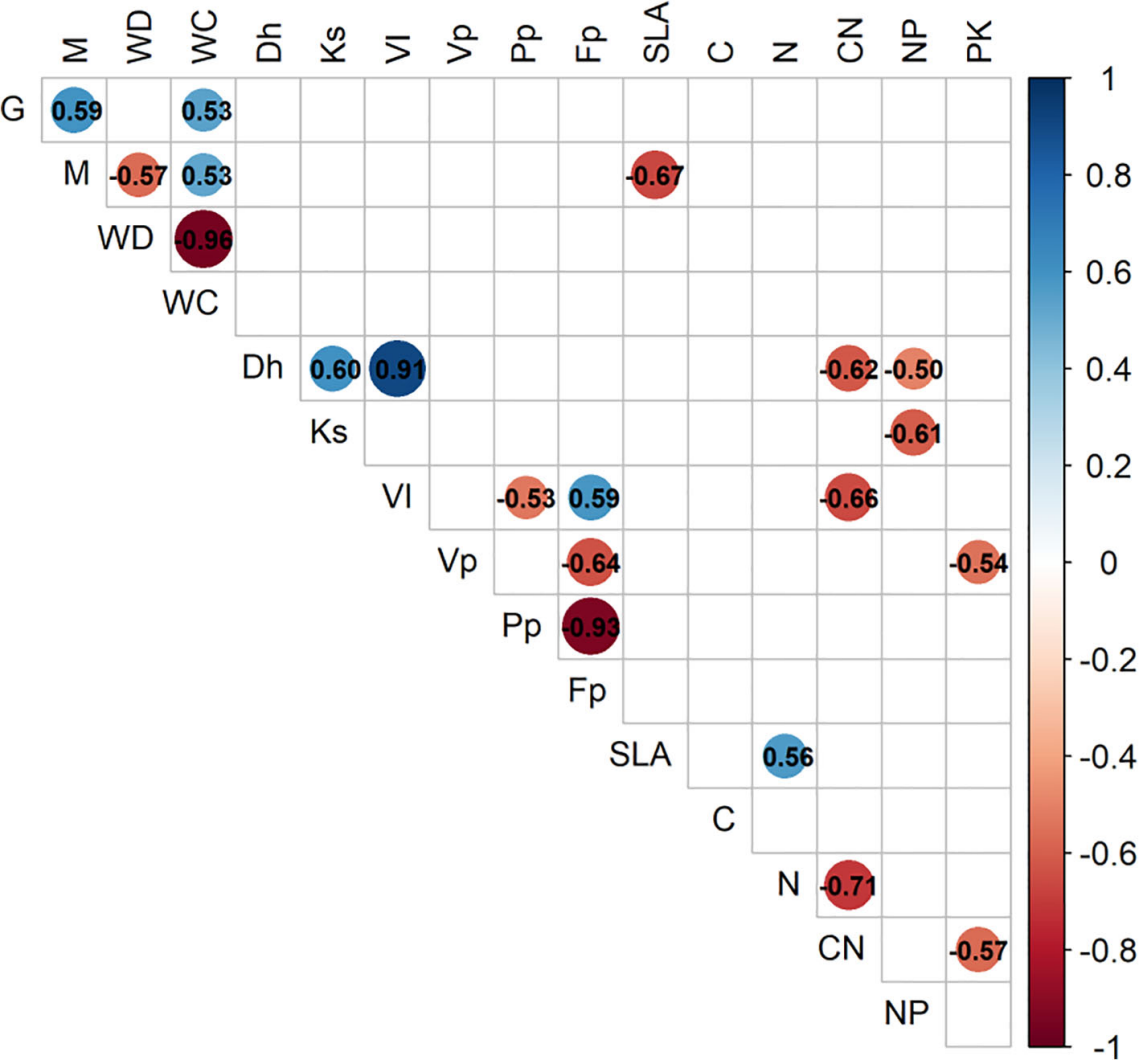
Pearson’s correlation matrix (p<0.05) of the interrelationships among 11 key plant functional traits and their relationship with growth (G) and mortality (M). Correlation strength and direction are indicated by color intensity and significant correlation statistics appear in the cells. Trait code abbreviations and units are described in **Table 2**. Trait values and other tree characteristics are shown in **Supplementary Table 2**.

**Figure 2:**
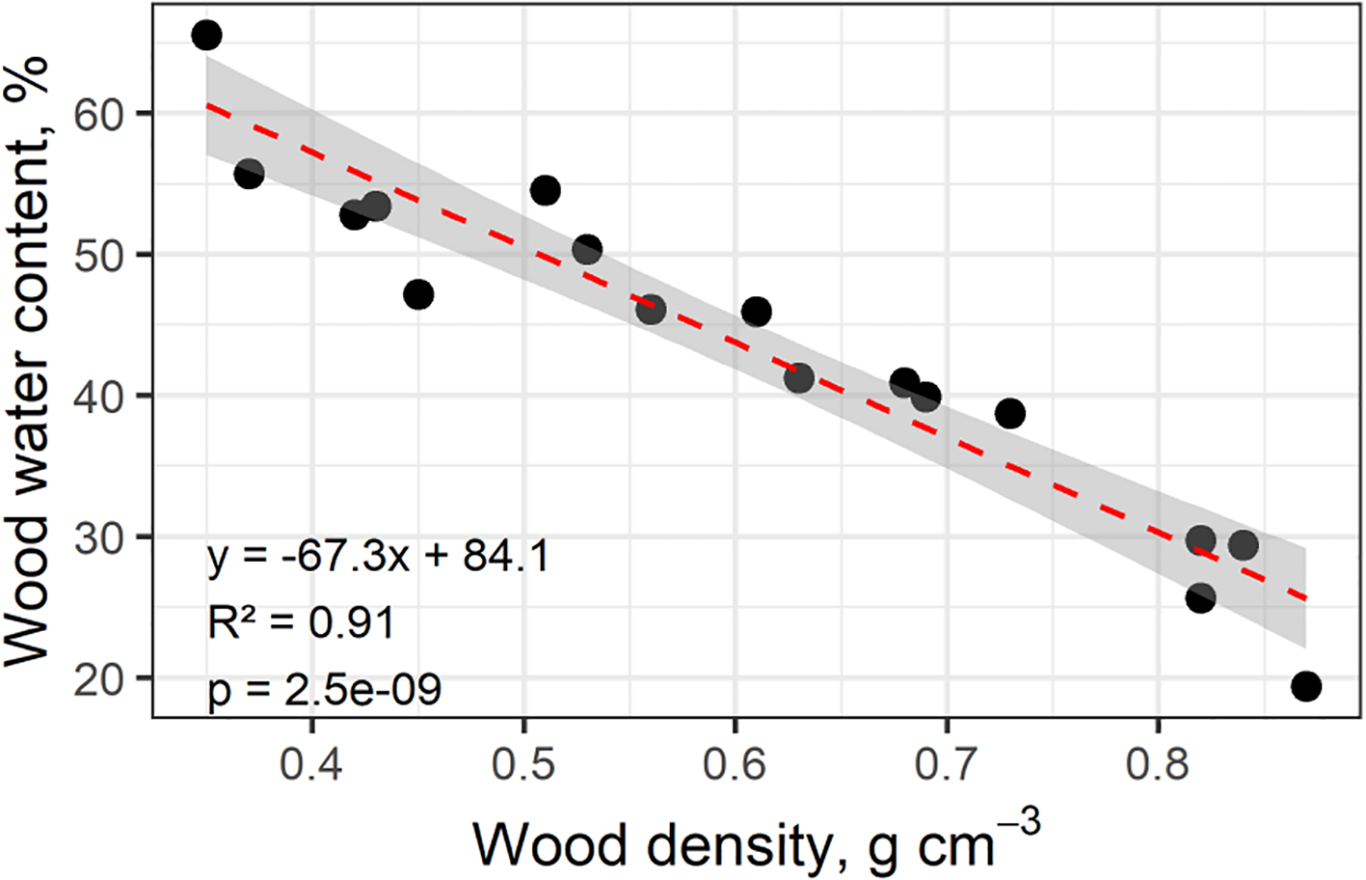
Saturated stem wood water content in relation to wood density for 17 individuals of different species of co-occurring upland Amazon trees (n=1). Water content was measured from rehydrated sub-samples collected at ~1.3 m after trees were felled.

To assess associations of plant trait relationships, we used a PCA of 11 key plant functional traits (PFTs), plus growth and mortality rates (**Figure 1**). For reference, we also explored a PCA of all 27 measured whole tree characteristics and functional traits (**Supplementary Table 1**). Wood density has a strong relationship with mechanical traits; therefore, it was expected that whole tree architectural characteristics would be positively correlated with wood density. However, none of the tree-level characteristics were significantly related to WD. Water content had the greatest correlation to wood density; WC strongly declined with increasing WD (**Figure 2**). Higher water content was correlated with faster growth and increased mortality rates (**Figure 1**). Mortality rate declined with increasing SLA (**Figure 1**). Considering the full correlation matrix, six other stem and leaf-level functional traits were related to wood density (WD) at the p<0.10 level (**Supplementary Table 1**). Wood density was negatively correlated to two of the stem hydraulic traits: sapwood fraction and sapwood depth. Wood density was correlated with half of the leaf functional traits; as WD increased, leaf size, leaf calcium and leaf magnesium levels decreased and the N:P ratio increased (**Supplementary Table 1**).

### 3.2 Relationships between functional traits

**Figure 3:**
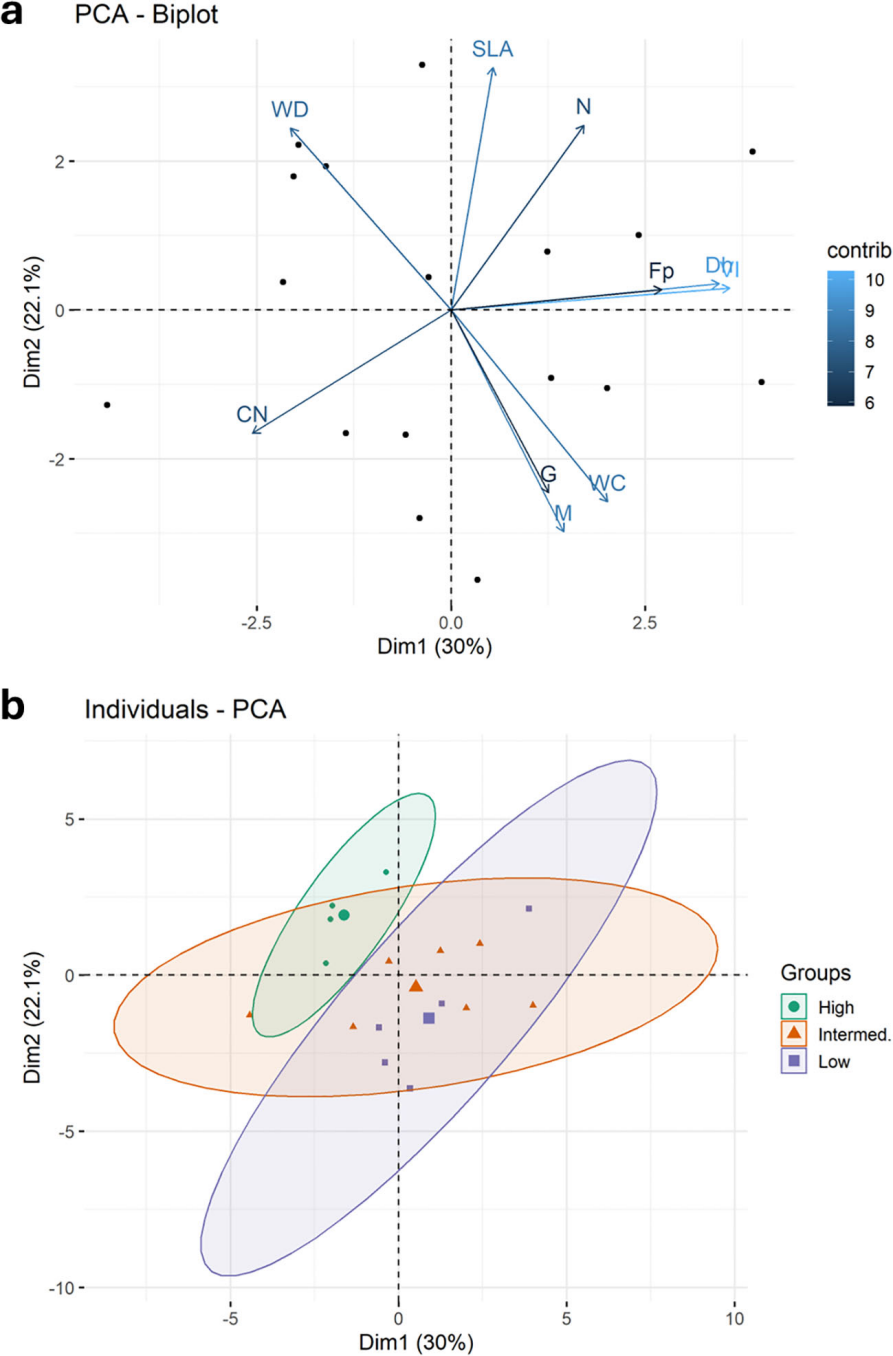
**(a)** Principal components analysis (PCA) of multivariate trait associations for 11 functional traits measured for 17 individuals of different species of co-occurring upland Amazon trees, including growth (G) and mortality (M) rates based on population scale demography. The 10 most significant loadings are indicated on the graph with arrows reflecting direction and strength of responses, and color intensity indicating contribution magnitude (WD, wood density; WC, wood water content; Dh, mean hydraulic diameter; VI, vulnerability index; Fp, fiber area; SLA, specific leaf area; CN, foliar carbon:nitrogen concentration; PK, foliar phosphorus:potassium concentration. **(b)** PCA grouping by wood density class; low WD < 0.5 g cm^-3^, intermediate WD 0.5 - 0.7 g cm^-3^, and high WD > 0.7 g cm^-3^. Average values for each wood density group are shown by the larger symbol). Also see **Figure 1**.

The first two components of the PCA explained 52% of the variation (**Figure 3**). The first axis explained 30% of the variation and was related to wood traits, with positive loading for mean vessel hydraulic diameter, hydraulic vulnerability index and wood fiber fraction. The second axis explained 22% of the variation and was related to wood and leaf traits, with negative loading for water content and foliar C:N and positive loading for wood density, specific leaf area and foliar N. Lower foliar C:N and N:P ratios were correlated with larger mean xylem hydraulic diameter (**Figure 1**).Growth and mortality rates mirrored WC and were in opposite direction of WD Faster growing species tended to have positive loading on the first axis and negative loading on the second axis, while slower growing species were opposite. There was full separation of loadings for the high- and low-density groups (**Figure 3B**).

Leaf size was correlated with increased wood water content. Foliar C and nutrient concentrations scaled with increased crown diameter and crown exposure (**Supplementary Table 1**). Foliar N and P were correlated and scaled with SLA. Foliar C:N and N:P declined as K increased and declined with increasing mean hydraulic diameter. As expected, larger vessels had greater mean hydraulic diameter, increased potential hydraulic conductivity and a higher vulnerability index. Trees with larger crowns were also correlated with higher potential hydraulic conductivity.

### 3.3 Potential trait linkages to demographics

**Figure 4:**
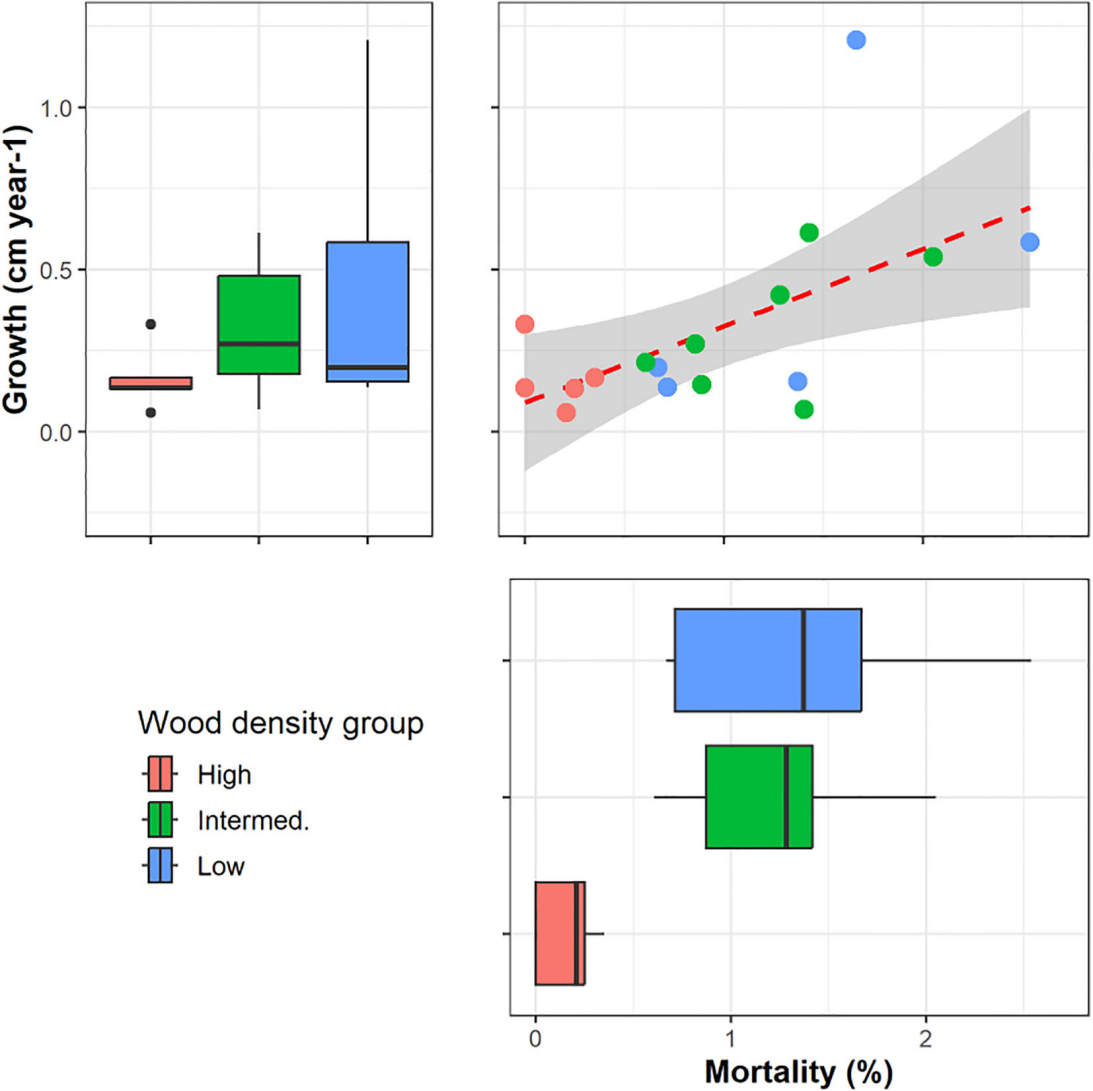
Stand level growth and mortality rates in relation to wood density class for the 17 species (n=1) harvested for this study. High wood density was associated with lower growth (p=0.06) and mortality (p=0.02). In the box plots, n=5–7 per WD class, the median is the thick line, the edges of the box are the lower and upper quantiles, Q1 and Q2, the whiskers are extreme values less than 1.5 of the interquartile range, and the points are outliers.

**Figure 5:**
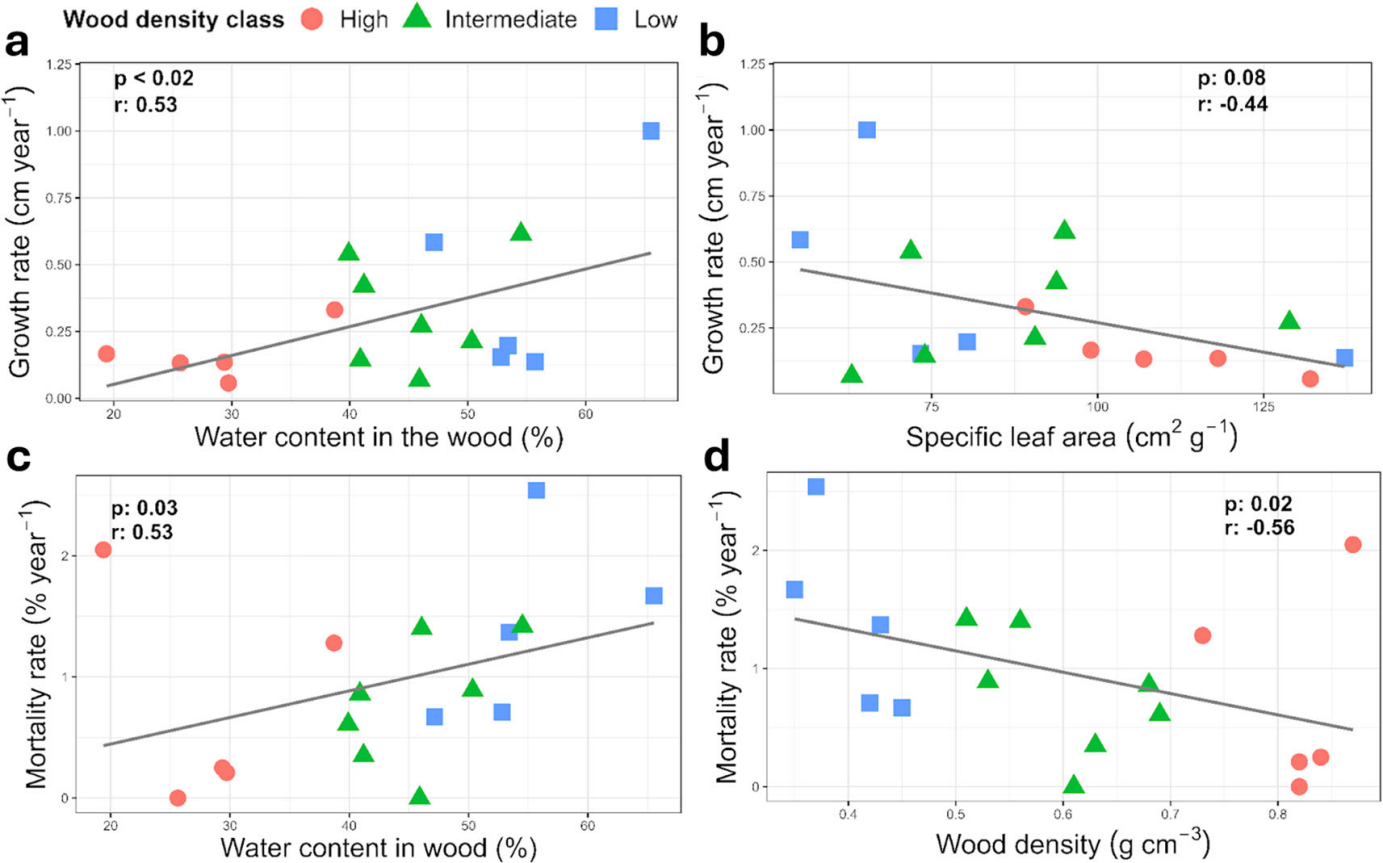
Pearson correlation relationships between select functional traits of the 17 individual harvested trees and stand level growth and mortality rates for the same species. Relative wood density classes are shown to explore potential interactions with the trait-growth relationships. **(a)** wood water content or **(b)** specific leaf area in relationship to growth rate, **(c)** wood water content or **(d)** wood density in relationship to mortality rate.

To explore potential linkages between tree functional traits and growth and mortality rates, we used Pearson correlation (r) of functional traits of each sampled individual against population level demography. Faster growth correlated with increased mortality rate (p=0.01; **Figure 4**). Increased wood density (**Figures 4**, **5D**) and lower wood water content (**Figures 5A, C**) correlated with reduced growth and mortality rates at the stand level. Increasing specific leaf area also correlated with reduced growth rates at the stand level (p=0.08, **Figure 5B**).

### 3.4 Axial variation of wood density and hydraulic traits

Initial regression analysis of stem traits across the wood density gradient showed that the only trait that varied significantly with tree height (e.g., between lower and upper axial positions in the stem) was sapwood area (p=0.05), which declined with height as expected. The decline in SWA between DBH and the base of the canopy for these trees was about 50%.

**Figure 6:**
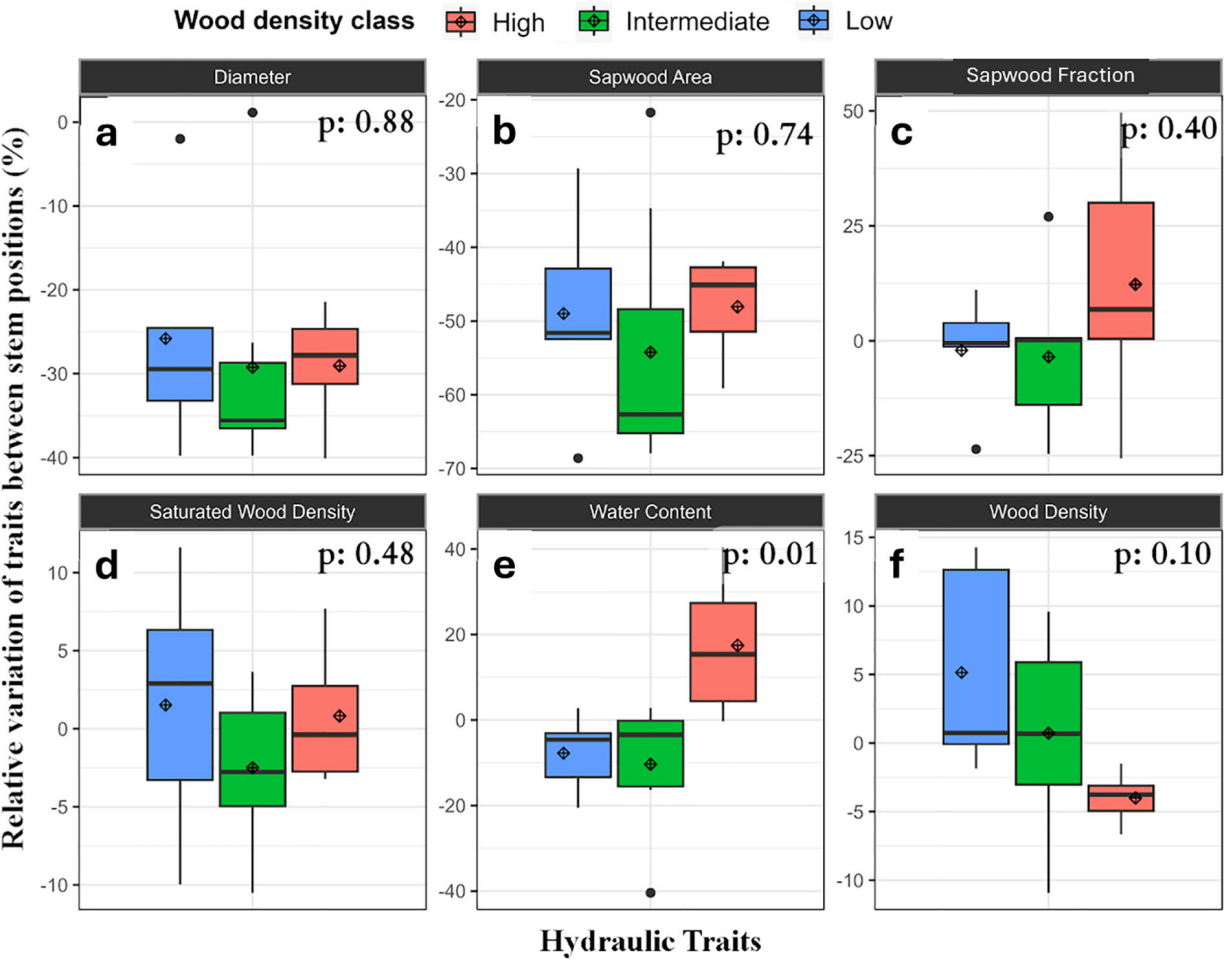
Relative variation (%) in hydraulic characteristics and traits between upper and lower stem sections; **(a)** diameter, **(b)** SWA, **(c)** SWF, **(d)** WD, **(e)** WC and **(f)** WD and hydraulic traits measured at different positions of the stem separated by wood density classes. Note that full anatomy measurements were only conducted at DBH. Relative variation between stem height positions are represented by (upper - lower)/lower) x 100% In the figure, the diamond represents the average and box plots components are as described in **Figure 4**.

Analyzing stem traits based on their wood density class revealed more information about possible wood density-dependent differences in upper and lower wood traits and revealed high variation in traits between wood density classes (**Figure 6**). That analysis indicated potential differences in water content and wood density with height. The highest wood density trees had a higher WC in the upper position, while low and intermediate density trees had similar WC at upper and lower positions (**Figure 6E**). There was also some evidence for a reduction in wood density with height for trees with the highest wood density trees showing a 5% decline in WD at the base of the canopy (**Figure 6F**).

### 3.5 Functional stem and leaf traits in relation to wood density

**Figure 7:**
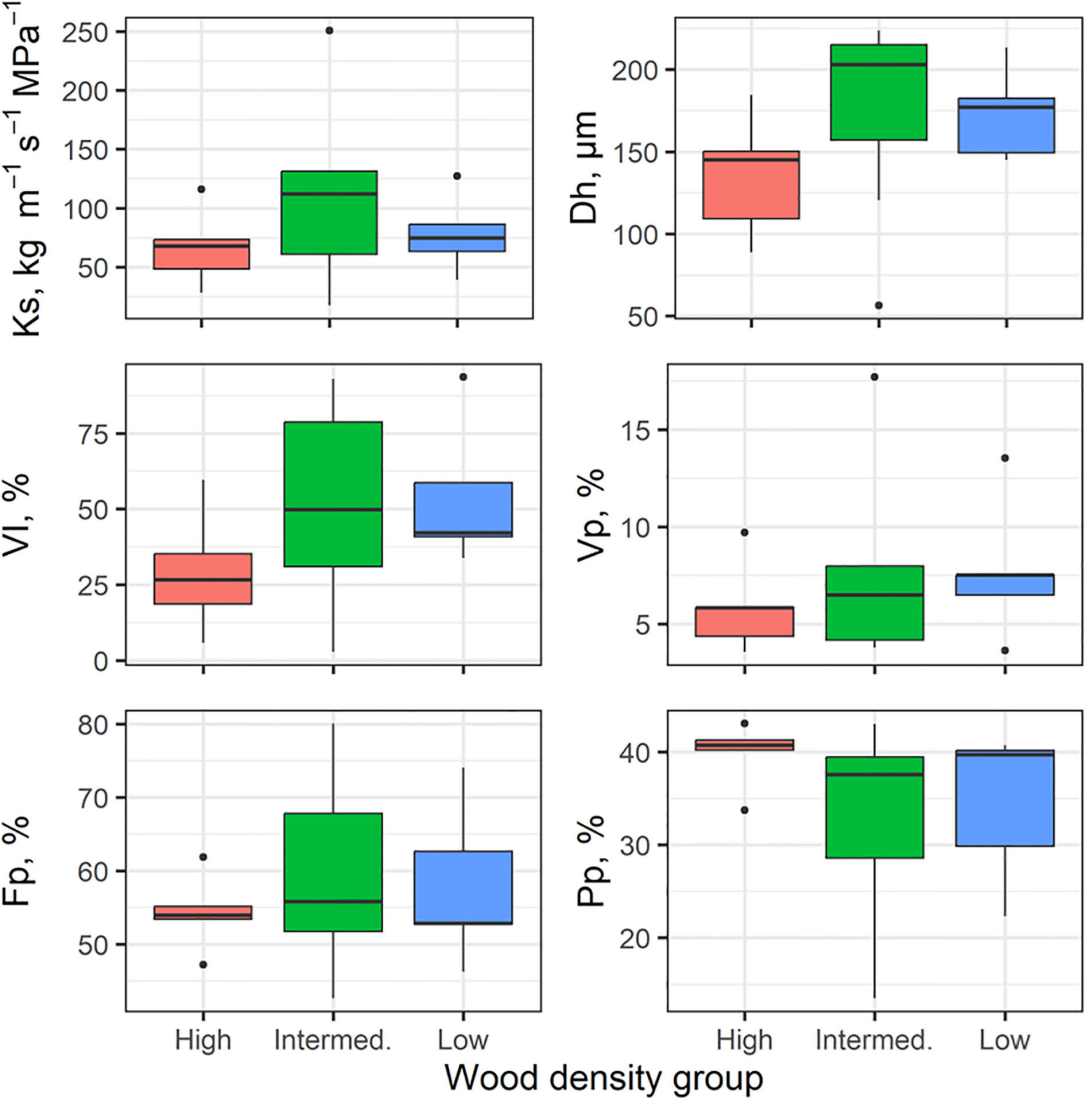
Variation in stem hydraulic traits across wood density class (high, intermediate, low), n=5–7 per WD. The box plots components are as described in **Figure 4**.

**Figure 8:**
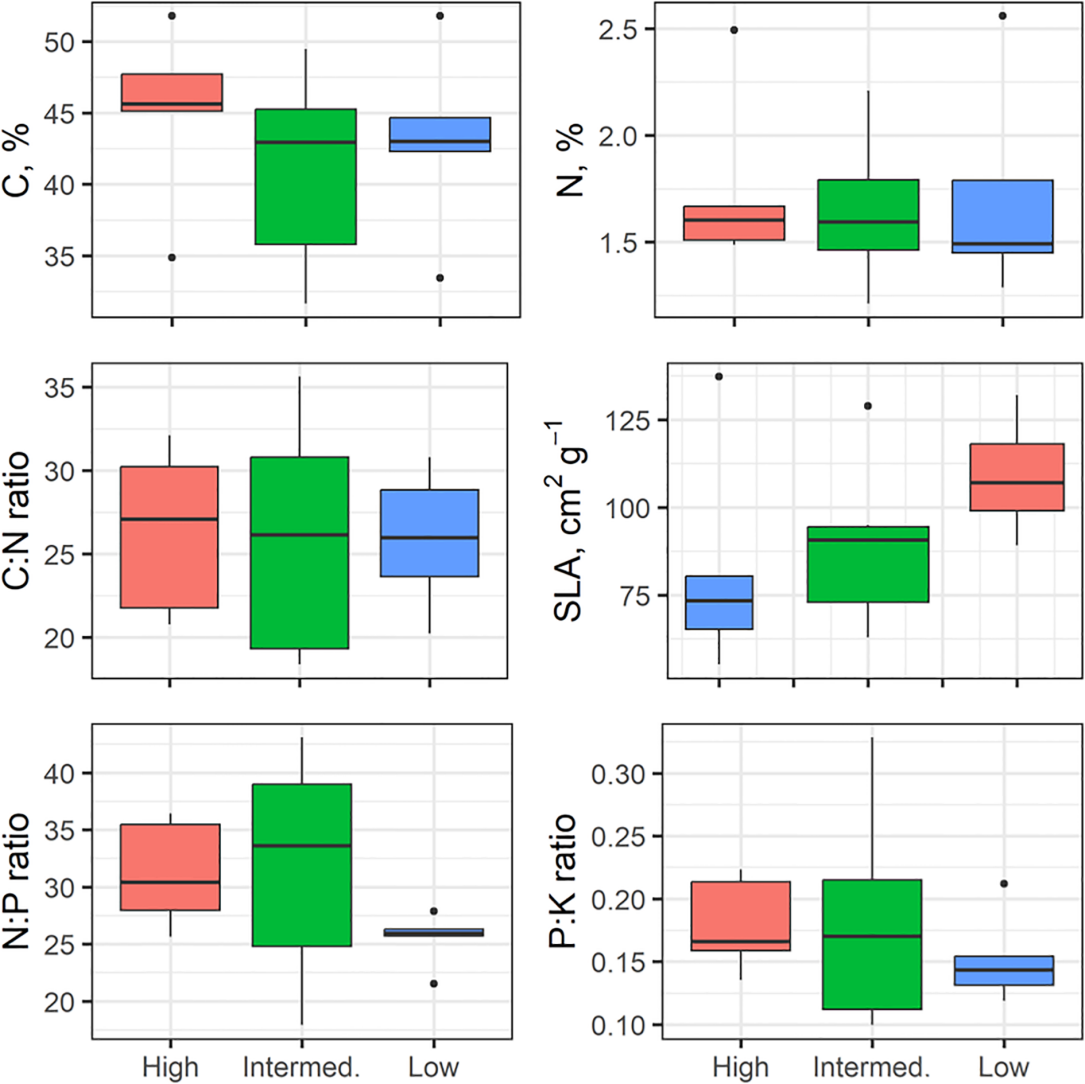
Variation in leaf traits across wood density class (high, intermediate, low), n=5–7 per WD class, +/- SD. The box plots components are as described in **Figure 4**.

There was wide variation for many of the stem and leaf traits in our 17 individuals (**Figures 7**, **8**). Based on non-parametric tests, no significant differences between wood density groups were found. Some traits tended to shift in magnitude with the highest wood density (e.g., smaller mean hydraulic diameter, Dh in **Figure 7**; increased SLA in **Figure 8**) but our lack of replication limited finding significant differences in these traits.

## 4 Discussion

### 4.1 No evidence for architectural trait linkage to wood density

Wood density (WD) is often associated with mechanical strength (Chave et al., 2009), and accordingly, architectural traits such as tree height and crown dimensions are expected to increase with WD (e.g., Iida et al., 2012; Francis et al., 2017). However, our results do not support that relationship and corroborate with previous studies suggesting that high WD does not necessarily confer a mechanical advantage for increased height (Aiba and Nakashizuka, 2009; Anten and Schieving, 2010; King et al., 2006). For instance, a study in the Central Amazon including 186 species across a broad diameter range found no relationship between WD and height (r = 0.10, p = 0.07; Nogueira et al., 2005). Similarly, a cross-continental comparison of species from Panama and Malaysia found no general relationship between WD and height, except in the smallest diameter class (< 2 cm) (Francis et al., 2017). While we found no relationship between WD and height, we did find increased stem length or height was associated with increased vessel diameter and lower vessel density, likely reflecting height dependence on water transport capacity independent of WD.

There is evidence that species with high WD have deeper crowns, possibly to optimize light interception (Augspurger and Kelly, 1984; Bohlman and O’Brien, 2006). Another study found crown width and depth scaled with WD across a wide diameter range, although not above 18 or 24 m height, respectively (Iida et al., 2012). However, crown dimensions, crown exposure and stem length (i.e., height of lower crown) were not related to WD in our study, opposite to what we expected. The lack of relationships may be due to the relatively low number of sample trees in this study or variable crown exposure levels that impacted resource acquisition and crown development. This may also be related to the narrow tree diameter (20 – 30 cm) or height range (16 – 28 m) used in this study, since the presence and strength of the WD tree architecture relationships may be linked to the ontogeny and size of the individual. One way to further explore these relationships would be to use other crown variables such as the shape or volume of the crown, or crown wood or leaf biomass, which could be approached with more objective techniques such as laser-based Lidar measurements (Gorgens et al., 2021). Additionally, in frequently wind-disturbed environments, the presence of multistemmed individuals has been interpreted as a structural adaptation to mechanical stress (Su et al., 2020). In the Central Amazon, wind disturbances have been shown to influence forest structure and dynamics by reducing the resilience of live tree biomass, particularly through the increased mortality of low WD species with shorter life spans (Magnabosco Marra et al., 2018). In this context, wind disturbance may play a role in shaping tree architecture independently of WD, and further investigation is warranted to understand how these factors interact.

### 4.2 Importance of wood density to hydraulic traits

Increasing WD leads to shifting trait-trade offs, such that plants with low WD are those that invest in high hydraulic transport capacity and water storage, while high wood density trees tend to have lower xylem conductivity but greater hydraulic safety (Santiago et al., 2018). In our study, xylem vessel size and potential hydraulic capacity were not significantly related to WD. Yet, note that the correlation values of vessel size-related characteristics Vd, Dh, Kp and Vp declined with WD, and values of vessel spatial distribution characteristics VD and VG increased with WD, in agreement with the signs of the hypothesized relationships. The low number of species used in this study (as a tradeoff to the large number of traits measured) likely limited some findings of significance. Measurements of fewer traits across a larger sample size is suggested for future research. Trait trade-offs also necessitate targeting a larger sample size. Using a global database of P50 and *Kp*, Gleason et al. (2016) demonstrated that it is not possible to have both high efficiency and high safety in plant hydraulic systems. However, many species have low efficiency and low safety, indicating the need to consider other trait tradeoffs and linkages to plant hydraulics, including investment in mechanical strength (e.g., fibers) and water or non-structural carbohydrate storage in parenchyma cells (Bittencourt et al., 2016).

Indeed, while we highlight hydraulic characteristics, wood has additional key functions related to biomechanical support, carbon assimilation (for green stems and twigs) and storage (Pratt and Jacobsen, 2017; Rungwattana and Hietz, 2018). Higher WD in our trees may reflect a greater investment in mechanical safety (higher proportion of heartwood, fibers and fiber wall thickness). Yet the lack of correlation of anatomical traits (Vp, Fp, Vp) with wood density found in our study corroborates with Ziemińska et al. (2013) indicating that vessel size or xylem cell types alone are not the main regulators of WD. Rather, WD is dependent on a variety of traits.

#### 4.2.1 Importance of wood water content

Water content of saturated samples may indicate the potential for water storage in the xylem (e.g., Goldstein et al., 1998). In our study, water content increased with sapwood fraction (SWF) and declined with wood density (**Supplementary Table 1**). WC also correlated with stand level growth rates (**Figure 3**). As stored water can be used to ensure water supply to the canopy in the early hours during the day or in short periods of water deficit (Goldstein et al., 1998; Scholz et al., 2011; van der Sande et al., 2015), stored water, along soil water is a key component of the vegetation water budget and thus carbon uptake. Higher wood WC of the lower WD trees as found in our study may help buffer daily development of water stress in these more acquisitive, faster growing trees. Similar to how P50 and *Kp* can act as environmental filters for the establishment of species (Oliveira et al., 2019; Brum et al., 2023), wood water content and water storage are key features that influence water use patterns and hydraulic traits of tropical species (Goldstein et al., 1998).

#### 4.2.2 Importance of sapwood area

Sapwood area is a key parameter used to understand and scale whole tree water use (Meinzer et al., 2003, 2005). The range of sapwood areas found in this study varied 2-fold despite only a 1.5-fold difference in tree diameter. Results were similar to those found in this and other tropical forests, and partially reflect the calculation of sapwood area, which includes a squared term. We previously reported active sapwood areas ranging from 202 to 1721 cm^2^ (8.5-fold) for dominant trees (DBH = 30 – 114 cm; 3.8-fold) in a nearby study in the Central Amazon (Spanner et al., 2022). Other studies in the Amazon found a 7.7-fold range in sapwood area for a 4.3-fold range of diameters in French Guiana (Granier et al., 1996) and 24.8-fold range of sapwood area for a 6.6-fold range of diameters in Venezuela (Anhuf et al., 1999). The wide range reflects the extreme diversity of tree species, hydraulic traits and size distribution in tropical forests (e.g., Cardoso et al., 2017). The variation in sapwood area for trees in the same size cohort as our study reflects the degree of difficulty for using these data for scaling from tree to stand level, e.g., for use in estimating total stand sap flow and transpiration (Spanner et al., 2022). Scaling to the stand level in these diverse stands may be more successful when considering a larger diameter range where species-specific differences are overshadowed by size dependence (e.g., Meinzer et al., 2005).

#### 4.2.3 No evidence for shifts in wood density or hydraulic traits with height

Across the 17 trees sampled, we found no significant differences in WD or wood hydraulic traits sampled between the lower and upper stem positions. This contrasts with a larger study conducted in the Central Amazon with 186 species that reported a general decrease in WD from the base to the crown (Nogueira et al., 2005). In that study, 87% of the individuals showed a reduction in WD with height— up to 57% in some cases—while others exhibited increases of up to 24%, underscoring the considerable variability in vertical WD patterns among species in upland Amazonian forests. The authors also noted that failing to account for this height-dependent variation could result in an overestimation of mean WD by approximately 5%. It is important to consider that their study encompassed a wide range of tree sizes (5 – 122 cm DBH), whereas our dataset focused on a narrower size range, which may limit the expression of these vertical trends.

Although no overall variation in wood traits was observed between stem positions across the WD gradient, some patterns emerged when individuals were grouped into WD classes. In trees with low and intermediate WD, we observed a modest decrease in the proportion of active xylem to sapwood area with height (-2% to -3.5%, respectively). In contrast, high WD trees exhibited an increase of approximately +12% (**Figure 6**). Similar patterns were observed in water content which, along with sapwood area, influences water storage and capacitance(e.g., Goldstein et al., 1998; Lira-Martins et al., 2022). These findings suggest potential trait plasticity in water storage, not only across individuals but also within the same tree, reflecting carbon allocation strategies that optimize the balance between water transport and hydraulic safety (Baas et al., 2004; Chave et al., 2009). While these observations were not significant, it does point to the need for more expansive measurements axially within the trees across a greater number of samples than used here in this study. Regarding the relationship between WD and canopy height, the evaluation of these traits at the community level—particularly along stronger environmental gradients such as disturbance regimes, edaphic variation, or climatic conditions—can potentially reveal clearer stand-level patterns. For example, at community-level WD tends to be higher, canopy height shorter, and mortality rates lower in drier or edaphically drier environments when assessed across broad gradients (e.g., Vargas et al., 2025). Such patterns suggest that trait–environment relationships may be more pronounced at larger spatial or ecological scales than within-individual comparisons allow as observed by this study.

### 4.3 Wood density and trait-trait tradeoffs

The relationship between wood density and tree traits can be considered as an indication of plant resource acquisition, where trees with higher wood densities are conservative species with slower growth rates, and trees with lower wood density are acquisitive species with faster growth rates (Santiago et al., 2018). In our sample trees, the correlations between WD and population demographic rates are consistent with these resource acquisition traits. More acquisitive species were also expected to have greater foliar nutrient concentrations, yet in this study there were no differences in foliar phosphorus or nitrogen between growth rate classes. However, foliar calcium and magnesium concentrations declined with increasing wood density. This could indicate more active root growth resulting in greater passive ion uptake rates that depend on new unsuberized roots (greater uptake rates) or increased transpiration rates (Mengel and Kirkby, 2001; Ahmed et al., 2023) or increased demand by the canopy, all of which are in agreement with an acquisitive resource response.

We also found leaf size decreased with increased WD. This also suggests that lower wood density species are generally associated with the fast-growing characteristic, with larger leaves (e.g., *Pouteria* and *Cecropia* genera), generally associated with higher photosynthetic rates (Slot and Winter, 2017) and monolayer leaf arrangement (Poorter et al., 2006). To a lesser extent, specific leaf area tended to increase with wood density (r=0.32) and decline with growth rate, as seen in other studies such as a tropical rainforest in Australia (Gray et al., 2019), which could indicate less photosynthetic capacity if foliar nutrient concentrations also decline with SLA. However, we found the opposite, with increasing concentrations of foliar N and P with increasing SLA. This suggests that growth rates were not solely dependent on foliar nutrient content, which may point to other factors, such as co-dependence of hydraulic controls on productivity associated with reduced hydraulic conductivity for slow growing trees, thereby affecting stomatal conductance and subsequent photosynthetic carbon uptake.

### 4.4 Trait correlations with growth and mortality

As expected, lower wood density was correlated with higher growth and mortality rates. This agrees with earlier results found across neotropical rainforests in Mexico, Panama and Bolivia (Poorter et al., 2008; Wright et al., 2010), and tropical rainforests in Venezuela and Brazil (Chao et al., 2008; Aleixo et al., 2019). In the latter study, growth rate and wood density together could be used to model mortality across species. Size was another predictor in that study, although this depended on location. In our study, crown exposure reflects more dominant trees within our narrow 20 – 30 cm diameter class, and the negative correlation of canopy exposure (Ce) with mortality indicates trees that have greater resource availability may be more successful. Higher WD was also correlated to lower theoretical hydraulic conductivity and lower mortality in a large study in Barro Colorado Island (BCI), Panama (Hietz et al., 2017). However, in that study, potential hydraulic conductivity (*Kp*) was not correlated with mortality, similar to the results of our study, reinforcing the variability in trait relationships due to trait-trait tradeoffs. Even so, our PCA results showing negative loading for WD and positive loading for vessel diameter (Vd) and Kp, along with lower water content with higher WD support the assumption that trees with larger vessels, greater hydraulic efficiencies and higher water storage have a more acquisitive life-response resulting in faster growth, albeit at greater risk of mortality. Nonetheless, the wide variation in some traits (e.g., SLA and N) within growth classes indicates a large range of different responses for resource acquisition and success.

## 5 Conclusion

WD was more correlated with stem hydraulic and leaf functional traits than with whole tree architectural characteristics. While we found no relationship between WD and height, we did find vessel diameter increased with increased stem length or height, likely reflecting height dependence on water transport capacity independent of WD. Lower wood density was associated with increased leaf size, foliar base cations, stem water content and sapwood fraction, and lower foliar N:P, in agreement with the fast-slow plant economics spectrum. Growth and mortality rates were greater for the intermediate and low wood densities trees as expected. While the correlation between wood density and other traits was weak in this small sample size, classifying trees as functional groups based on wood density revealed differences in these leaf and wood anatomical traits. This supports the hypothesis that trees assemble along trait space dimensions along a wood density axis.

## License and permission statement

This manuscript has been authored by UT-Battelle, LLC under Contract No. DE-AC05-00OR22725 with the US Department of Energy. The United States Government retains and the publisher, by accepting the article for publication, acknowledges that the United States Government retains a non-exclusive, paid-up, irrevocable, world-wide license to publish or reproduce the published form of this manuscript, or allow others to do so, for United States Government purposes. The Department of Energy will provide public access to these results of federally sponsored research in accordance with the DOE Public Access Plan (http://energy.gov/downloads/doe-public-access-plan).

## Data Availability

The original contributions presented in the study are publicly available. The data can be found at the NGEE-Tropics project data archive and the DOE long-term repository, ESS-DIVE: https://data.ess-dive.lbl.gov/datasets/doi:10.15485/2998005.

## Supplementary material

Supplementary Table 1 Pearson correlation matrix of all tree measured tree characteristics and functional traits in respect to wood density and each other. Significant correlations at the p<0.05 level are shown in bold and at the p<0.10 level underlined. Tree characteristics and architectural traits are highlighted in green, stem wood traits in yellow and leaf traits in blue. Trait code abbreviations and units are described in **Table 2**.

Supplementary Table 2 Tree characteristics and functional trait measurements for 17 individual sample trees of different species. Population level growth and mortality data for specific species based on long term demography plots are included for reference. Trait code abbreviations and units are described in **Table 2**.

Supplementary Table 3 Principal Component Analysis (PCA) loadings for select key functional traits, plus population level growth (G) and mortality (M) rates. PCA differentiates the nature of variable contributions, where positive loadings suggest a positive correlation, and negative loadings a negative one. The magnitude of these loadings underscores the significance of each variable in constituting the principal components. Trait code abbreviations and units are described in **Table 2**.

Supplementary Figure 1 Detailed flowchart of sapwood area measurement process. This figure depicts the sequential steps involved in quantifying the active xylem area, commonly referred to as sapwood area, including **(A)** tree selection and felling, **(B)** precise extraction of samples, **(C)** immersion in acid fuchsin dye for enhanced visibility, **(D)** monitoring dye ascent to delineate sapwood, **(E)** and retrieval of discs for conductive area analysis.

Supplementary Figure 2 Example images of stem xylem anatomy. *Simarouba amara* and *Tachigali paniculate* had the fastest growth rates and *Lecythis pisonis* and *Minquartia guianensis* had the slowest growth rates.
