# Supplementary material for: Mortality correlates with tree functional traits across a wood density gradient in the Central Amazon
Source: Front Plant Sci. 2025 Oct 15;16:1572767. doi: 10.3389/fpls.2025.1572767 (PMC12569815; doi:10.3389/fpls.2025.1572767)
Supplement: Supplementary Table 1 — Pearson correlation matrix of all tree measured tree characteristics and functional traits in respect to wood density and each other. Significant correlations at the p<0.05 level are shown in bold and at the p<0.10 level underlined. Tree characteristics and architectural traits are highlighted in green, stem wood traits in yellow and leaf traits in blue. Trait code abbreviations and units are described in Table 2 . [file DataSheet1.docx]

# **Supplementary Material**

**Mortality correlates with tree functional traits across a wood density gradient in the Central Amazon**

*Valdiek da Silva Menezes^1^, Bruno O. Gimenez^1,2^, Cynthia L. Wright^3,4^, Niro Higuchi^1^, Claudete C. Nascimento^5^, Fernanda B. Barros^5^, Gustavo C. Spanner^1^, Jardel R. Rodrigues^1^, Nate McDowell^6,7^, Adam D. Collins^8^, Robinson I. Negrón-Juárez^9^, Jeffrey Q. Chambers^2,9^, Brent D. Newman^8^, Adriano José Nogueira Lima^1^, and Jeffrey M. Warren^3*^*

**Supplementary Table 1.** Pearson correlation matrix of all tree measured tree characteristics and functional traits in respect to wood density and each other. Significant correlations at the p<0.05 level are shown in bold and at the p<0.10 level underlined. Tree characteristics and architectural traits are highlighted in green, stem wood traits in yellow and leaf traits in blue. Trait code abbreviations and units are described in Table 2.

|  | **DBH** | **Ht** | **Sl** | **Cl** | **Cd** | **Ce** | **SWA** | **SWF** | **SWD** | **WD** | **WC** | **Vd** | **VD** | **VG** | **Dh** | **Kp** | **VI** | **Vp** | **Pp** | **Fp** | **LS** | **SLA** | **C** | **N** | **P** | **K** | **Ca** | **Mg** |
| --- | --- | --- | --- | --- | --- | --- | --- | --- | --- | --- | --- | --- | --- | --- | --- | --- | --- | --- | --- | --- | --- | --- | --- | --- | --- | --- | --- | --- |
| **Ht** | **-0.15** |  |  |  |  |  |  |  |  |  |  |  |  |  |  |  |  |  |  |  |  |  |  |  |  |  |  |  |
| **Sl** | **-0.04** | **0.84** |  |  |  |  |  |  |  |  |  |  |  |  |  |  |  |  |  |  |  |  |  |  |  |  |  |  |
| **Cl** | **-0.27** | **0.32** | **-0.24** |  |  |  |  |  |  |  |  |  |  |  |  |  |  |  |  |  |  |  |  |  |  |  |  |  |
| **Cd** | **0.25** | **-0.03** | **-0.08** | **0.06** |  |  |  |  |  |  |  |  |  |  |  |  |  |  |  |  |  |  |  |  |  |  |  |  |
| **Ce** | **0.54** | **0.34** | **0.12** | **0.34** | **0.29** |  |  |  |  |  |  |  |  |  |  |  |  |  |  |  |  |  |  |  |  |  |  |  |
| **SWA** | **0.38** | **0.05** | **0.05** | **-0.08** | **0.29** | **0.34** |  |  |  |  |  |  |  |  |  |  |  |  |  |  |  |  |  |  |  |  |  |  |
| **SWP** | **-0.33** | **0.17** | **0.05** | **0.17** | **0.02** | **0.00** | **0.72** |  |  |  |  |  |  |  |  |  |  |  |  |  |  |  |  |  |  |  |  |  |
| **SWD** | **0.01** | **0.08** | **-0.04** | **-0.27** | **0.25** | **0.54** | **0.89** | **0.91** |  |  |  |  |  |  |  |  |  |  |  |  |  |  |  |  |  |  |  |  |
| **WD** | **0.39** | **0.01** | **-0.06** | **0.13** | **0.17** | **0.32** | **-0.23** | **-0.55** | **-0.49** |  |  |  |  |  |  |  |  |  |  |  |  |  |  |  |  |  |  |  |
| **WC** | **-0.44** | **0.02** | **0.01** | **0.04** | **-0.29** | **-0.31** | **0.08** | **0.50** | **0.34** | **-0.89** |  |  |  |  |  |  |  |  |  |  |  |  |  |  |  |  |  |  |
| **Vd** | **-0.33** | **0.48** | **0.41** | **0.16** | **0.23** | **0.11** | **0.11** | **0.30** | **0.24** | **-0.39** | **0.17** |  |  |  |  |  |  |  |  |  |  |  |  |  |  |  |  |  |
| **VD** | **0.25** | **-0.47** | **-0.49** | **0.01** | **-0.10** | **-0.02** | **-0.19** | **-0.38** | **-0.33** | **0.34** | **-0.17** | **-0.82** |  |  |  |  |  |  |  |  |  |  |  |  |  |  |  |  |
| **VG** | **0.36** | **-0.19** | **0.02** | **-0.38** | **-0.08** | **-0.02** | **0.12** | **-0.11** | **-0.08** | **0.09** | **-0.01** | **-0.58** | **0.51** |  |  |  |  |  |  |  |  |  |  |  |  |  |  |  |
| **Dh** | **-0.32** | **0.52** | **0.47** | **0.13** | **0.21** | **0.11** | **0.08** | **0.27** | **0.21** | **-0.39** | **0.17** | **0.99** | **-0.81** | **-0.51** |  |  |  |  |  |  |  |  |  |  |  |  |  |  |
| **Kp** | **-0.20** | **0.37** | **0.30** | **0.17** | **0.49** | **0.15** | **-0.08** | **-0.04** | **-0.11** | **-0.22** | **0.03** | **0.72** | **-0.36** | **-0.37** | **0.73** |  |  |  |  |  |  |  |  |  |  |  |  |  |
| **VI** | **-0.28** | **0.43** | **0.36** | **0.16** | **-0.16** | **0.04** | **0.12** | **0.34** | **0.33** | **-0.38** | **0.24** | **0.81** | **-0.71** | **-0.36** | **0.82** | **0.29** |  |  |  |  |  |  |  |  |  |  |  |  |
| **Vp** | **-0.12** | **-0.32** | **-0.21** | **-0.20** | **0.11** | **-0.07** | **-0.04** | **0.02** | **-0.10** | **-0.16** | **0.27** | **-0.35** | **0.57** | **0.50** | **-0.34** | **0.02** | **-0.43** |  |  |  |  |  |  |  |  |  |  |  |
| **Pp** | **0.17** | **0.04** | **-0.14** | **0.25** | **0.50** | **0.42** | **-0.04** | **-0.13** | **-0.18** | **0.32** | **-0.28** | **-0.22** | **0.20** | **0.09** | **-0.23** | **0.10** | **-0.53** | **0.32** |  |  |  |  |  |  |  |  |  |  |
| **Fp** | **-0.09** | **0.09** | **0.20** | **-0.13** | **-0.44** | **-0.31** | **0.05** | **0.10** | **0.18** | **-0.20** | **0.12** | **0.31** | **-0.38** | **-0.27** | **0.32** | **-0.09** | **0.59** | **-0.64** | **-0.93** |  |  |  |  |  |  |  |  |  |
| **LS** | **-0.13** | **-0.05** | **-0.20** | **0.27** | **-0.36** | **-0.03** | **0.11** | **0.26** | **0.33** | **-0.56** | **0.54** | **0.25** | **-0.23** | **-0.27** | **0.24** | **-0.03** | **0.44** | **-0.24** | **-0.40** | **0.41** |  |  |  |  |  |  |  |  |
| **SLA** | **0.31** | **0.01** | **-0.14** | **0.31** | **0.11** | **0.40** | **-0.24** | **-0.49** | **-0.29** | **0.32** | **-0.35** | **0.15** | **0.03** | **-0.18** | **0.17** | **0.31** | **0.11** | **-0.29** | **-0.06** | **0.16** | **0.35** |  |  |  |  |  |  |  |
| **C** | **0.52** | **-0.26** | **-0.42** | **0.22** | **0.45** | **0.49** | **0.26** | **-0.07** | **0.16** | **0.08** | **-0.07** | **-0.17** | **0.25** | **-0.15** | **-0.21** | **-0.03** | **-0.23** | **0.01** | **0.26** | **-0.21** | **0.26** | **0.40** |  |  |  |  |  |  |
| **N** | **0.42** | **0.19** | **0.17** | **0.04** | **0.11** | **0.45** | **0.16** | **-0.12** | **0.07** | **-0.03** | **-0.03** | **0.30** | **-0.26** | **0.01** | **0.32** | **0.05** | **0.48** | **-0.23** | **-0.12** | **0.19** | **0.47** | **0.56** | **0.43** |  |  |  |  |  |
| **P** | **0.34** | **0.25** | **0.19** | **0.09** | **0.25** | **0.52** | **0.29** | **0.05** | **0.22** | **-0.33** | **0.20** | **0.45** | **-0.33** | **-0.03** | **0.48** | **0.39** | **0.41** | **-0.16** | **-0.03** | **0.09** | **0.56** | **0.57** | **0.47** | **0.82** |  |  |  |  |
| **K** | **0.23** | **0.11** | **0.09** | **0.02** | **0.50** | **0.40** | **0.12** | **-0.03** | **0.04** | **-0.34** | **0.28** | **0.29** | **-0.14** | **-0.02** | **0.30** | **0.52** | **0.05** | **0.21** | **0.24** | **-0.27** | **0.25** | **0.37** | **0.60** | **0.48** | **0.76** |  |  |  |
| **Ca** | **-0.60** | **0.04** | **0.20** | **-0.25** | **-0.24** | **-0.42** | **-0.07** | **0.32** | **0.08** | **-0.61** | **0.47** | **0.46** | **-0.28** | **-0.23** | **0.44** | **0.25** | **0.39** | **0.22** | **-0.28** | **0.15** | **0.03** | **-0.43** | **-0.50** | **-0.18** | **-0.12** | **-0.14** |  |  |
| **Mg** | **0.15** | **-0.09** | **0.15** | **-0.48** | **0.11** | **0.15** | **0.36** | **0.26** | **0.24** | **-0.54** | **0.37** | **0.06** | **0.08** | **0.30** | **0.07** | **0.22** | **-0.11** | **0.53** | **0.09** | **-0.27** | **0.00** | **-0.19** | **0.13** | **0.07** | **0.36** | **0.52** | **0.42** |  |
| **N:P** | **-0.07** | **-0.13** | **-0.06** | **-0.11** | **-0.34** | **-0.39** | **-0.15** | **-0.09** | **-0.17** | **0.47** | **-0.32** | **-0.53** | **0.39** | **0.16** | **-0.54** | **-0.61** | **-0.25** | **0.07** | **-0.16** | **0.10** | **-0.41** | **-0.39** | **-0.28** | **-0.39** | **-0.80** | **-0.71** | **-0.06** | **-0.42** |

**Supplementary Table 2.** Tree characteristics and functional trait measurements for 17 individual sample trees of different species. Population level growth and mortality data for specific species based on long term demography plots is included for reference. Trait code abbreviations and units are described in Table 2.

| **id** | **Species** | **Growth rate** | **Mortality rate** | **Wood density class** | **DBH** | **Ht** | **Sl** | **Cl** | **Cd** | **Ce** | **SWA** | **SWF** | **WD** | **WC** | **Vd** | **VD** | **VG** | **Dh** | **Kp** | **Vp** | **Pp** | **Fp** | **LS** | **SLA** | **C** | **N** | **P** | **K** | **Ca** | **Mg** | **N:P** |
| --- | --- | --- | --- | --- | --- | --- | --- | --- | --- | --- | --- | --- | --- | --- | --- | --- | --- | --- | --- | --- | --- | --- | --- | --- | --- | --- | --- | --- | --- | --- | --- |
| 1 | *Simarouba amara* | 1.21 | 1.67 | Low | 19.7 | 23.1 | 12.28 | 10.8 | 4.5 | 3 | 238 | 99 | 0.35 | 65.5 | 136 | 3.4 | 1.4 | 145 | 39.3 | 7.5 | 39.7 | 52.8 | 411.5 | 65.3 | 43.0 | 14.9 | 0.6 | 4.4 | 3.7 | 0.6 | 25.7 |
| 2 | *Virola pavonis* | 0.15 | 1.37 | Low | 28 | 16.2 | 10.6 | 5.0 | 7.7 | 3 | 458 | 95 | 0.42 | 52.8 | 158 | 2.8 | 2.3 | 182 | 63.3 | 7.5 | 29.9 | 62.6 | 54.6 | 73.4 | 42.3 | 17.9 | 0.7 | 4.5 | 4.8 | 1.2 | 25.9 |
| 3 | *Sterculia excelsa* | 0.20 | 0.67 | Low | 19.1 | 20.5 | 14.55 | 6.0 | 4.3 | 3 | 193 | 81 | 0.43 | 53.4 | 171 | 4.1 | 1.5 | 177 | 127 | 13.5 | 40.2 | 46.3 | 124.2 | 80.3 | 33.4 | 12.9 | 0.5 | 4.1 | 6.9 | 1.4 | 26.3 |
| 4 | *Pourouma myrmecophila* | 0.58 | 2.54 | Low | 25 | 18.1 | 12.6 | 5.5 | 8 | 2 | 309 | 71 | 0.45 | 47.2 | 154 | 4.6 | 1.3 | 149 | 86.2 | 6.5 | 40.7 | 52.8 | 148.6 | 55.2 | 44.7 | 14.5 | 0.5 | 3.6 | 4.5 | 0.9 | 27.9 |
| 5 | *Jacaranda copaia* | 0.14 | 0.71 | Low | 26.5 | 20.2 | 11 | 9.2 | 4.5 | 4 | 455 | 100 | 0.37 | 55.7 | 183 | 2.0 | 1.2 | 213 | 74.4 | 3.6 | 22.3 | 74.0 | 870.8 | 137.2 | 51.8 | 25.6 | 1.2 | 5.6 | 3.1 | 0.9 | 21.5 |
| 6 | *Tachigali paniculata* | 0.61 | 1.43 | Intermediate | 19.4 | 21.9 | 16.9 | 6.0 | 5 | 1 | 145 | 58 | 0.51 | 54.5 | 188 | 2.4 | 1.4 | 224 | 131 | 6.5 | 13.5 | 80.0 | 197.9 | 95.0 | 31.6 | 16.5 | 0.5 | 2.5 | 5.8 | 0.5 | 33.6 |
| 7 | *Guatteria olivacea* | 0.54 | 2.05 | Intermediate | 22 | 25.9 | 15.8 | 10.1 | 5 | 4 | 326 | 99 | 0.69 | 39.9 | 183 | 2.0 | 1.5 | 216 | 82.2 | 3.8 | 30.1 | 66.2 | 38.0 | 71.9 | 36.0 | 13.8 | 0.4 | 1.7 | 3.5 | 0.4 | 36.2 |
| 8 | *Micropholis guyanensis* | 0.14 | 0.89 | Intermediate | 26.1 | 17.43 | 9.43 | 8.0 | 6 | 3 | 296 | 67 | 0.68 | 40.9 | 67 | 24 | 2.7 | 57 | 17.6 | 17.7 | 39.2 | 42.6 | 23.1 | 74.0 | 47.4 | 15.5 | 0.4 | 3.6 | 3.3 | 1.0 | 43.1 |
| 9 | *Scleronema micranthum* | 0.27 | 0.86 | Intermediate | 25.4 | 24 | 14 | 10.0 | 10.5 | 4 | 249 | 58 | 0.56 | 46.1 | 192 | 5.2 | 1.2 | 194 | 251 | 8.0 | 43.0 | 49.0 | 116.2 | 128.9 | 49.5 | 15.9 | 0.9 | 8.4 | 2.4 | 0.9 | 17.9 |
| 10 | *Goupia glabra* | 0.33 | 0.00 | High | 26.6 | 19.4 | 10.6 | 8.8 | 10 | 5 | 509 | 98 | 0.73 | 38.7 | 159 | 6.0 | 1.3 | 145 | 116 | 9.7 | 43.1 | 47.2 | 35.1 | 89.1 | 47.7 | 14.9 | 0.6 | 3.7 | 2.9 | 0.8 | 25.6 |
| 11 | *Hevea guianensis* | 0.21 | 0.61 | Intermediate | 28.1 | 28.5 | 22.4 | 6.1 | 7.5 | 5 | 456 | 87 | 0.53 | 50.3 | 161 | 3.5 | 2.5 | 203 | 112 | 8.0 | 37.6 | 54.5 | 78.2 | 90.6 | 42.9 | 22.1 | 1.1 | 6.4 | 3.4 | 1.4 | 20.3 |
| 12 | *Pouteria venosa* | 0.17 | 0.35 | High | 28.5 | 21.37 | 14.8 | 6.6 | 5.5 | 3 | 276 | 51 | 0.87 | 19.4 | 103 | 5.9 | 2.5 | 109 | 28.4 | 5.9 | 40.8 | 53.4 | 49.8 | 99.0 | 34.9 | 16.0 | 0.4 | 2.1 | 0.6 | 0.4 | 36.4 |
| 13 | *Licaria martiniana* | 0.07 | 1.40 | Intermediate | 26.7 | 23.5 | 17.15 | 6.0 | 4.5 | 3 | 433 | 93 | 0.61 | 45.9 | 127 | 5.1 | 1.1 | 120 | 39.2 | 3.8 | 27.1 | 69.4 | 42.9 | 63.0 | 43.2 | 12.1 | 0.3 | 2.4 | 3.9 | 0.9 | 41.8 |
| 14 | *Minquartia guianensis* | 0.06 | 0.21 | High | 28.4 | 17.46 | 9.3 | 8.2 | 4 | 4 | 138 | 23 | 0.82 | 29.7 | 94 | 16 | 1.8 | 89 | 48.5 | 4.4 | 33.7 | 61.9 | 140.1 | 132.0 | 45.6 | 15.1 | 0.5 | 2.4 | 2.2 | 0.7 | 27.9 |
| 15 | *Lecythis pisonis* | 0.14 | 0.00 | High | 29.2 | 22.5 | 15.5 | 7.0 | 7.5 | 5 | 175 | 31 | 0.84 | 29.4 | 162 | 2.8 | 1.2 | 184 | 73.4 | 5.8 | 40.2 | 53.9 | 59.1 | 118.1 | 51.8 | 24.9 | 0.8 | 6.1 | 2.6 | 0.6 | 30.4 |
| 16 | *Eschweilera coriacea* | 0.13 | 0.25 | High | 21.5 | 21.26 | 11.26 | 10.0 | 8.8 | 3 | 273 | 83 | 0.82 | 25.6 | 150 | 4.4 | 1.1 | 150 | 67.5 | 3.6 | 41.3 | 55.2 | 40.1 | 107.0 | 45.1 | 16.7 | 0.5 | 2.8 | 3.1 | 0.4 | 35.4 |
| 17 | *Inga paraensis* | 0.42 | 1.28 | Intermediate | 24.9 | 26.97 | 16.7 | 10.3 | 5.5 | 4 | 307 | 74 | 0.63 | 41.2 | 197 | 2.6 | 1.2 | 214 | 132.1 | 4.6 | 39.6 | 55.8 | 135.7 | 93.8 | 35.6 | 19.4 | 0.7 | 2.0 | 5.6 | 0.7 | 29.4 |

**Supplementary Table 3.** Principal Component Analysis (PCA) loadings for select key functional traits, plus population level growth (G) and mortality (M) rates. PCA differentiates the nature of variable contributions, where positive loadings suggest a positive correlation, and negative loadings a negative one. The magnitude of these loadings underscores the significance of each variable in constituting the principal components. Trait code abbreviations and units are described in Table 2.

|  | Dim 1 | Dim 2 | Dim 3 | Dim 4 | Dim 5 |
| --- | --- | --- | --- | --- | --- |
| G | 0.1571 | 0.3212 | 0.0057 | 0.2306 | 0.0128 |
| M | 0.2185 | 0.3792 | 0.1610 | 0.0692 | 0.0358 |
| WD | 0.3763 | 0.4500 | 0.0280 | 0.0539 | 0.0057 |
| WC | 0.3674 | 0.4885 | 0.0187 | 0.0669 | 0.0064 |
| Dh | 0.7579 | 0.0231 | 0.0967 | 0.0164 | 0.0013 |
| Ks | 0.1218 | <0.0001 | 0.5396 | 0.0010 | 0.0750 |
| VI | 0.8126 | 0.0350 | <0.0001 | 0.0142 | 0.0248 |
| Vp | 0.1986 | 0.2787 | 0.0876 | 0.0274 | 0.3026 |
| Pp | 0.4145 | 0.0135 | 0.2532 | 0.2674 | 0.0030 |
| Fp | 0.4785 | 0.0847 | 0.2694 | 0.1287 | 0.0275 |
| SLA | 0.0028 | 0.5574 | 0.1897 | 0.0896 | 0.0173 |
| CN | 0.3133 | 0.2193 | 0.0139 | 0.0329 | 0.3530 |
| NP | 0.1581 | 0.0018 | 0.6439 | 0.0027 | 0.0298 |
| PK | 0.1821 | 0.3361 | 0.0405 | 0.2153 | 0.0045 |


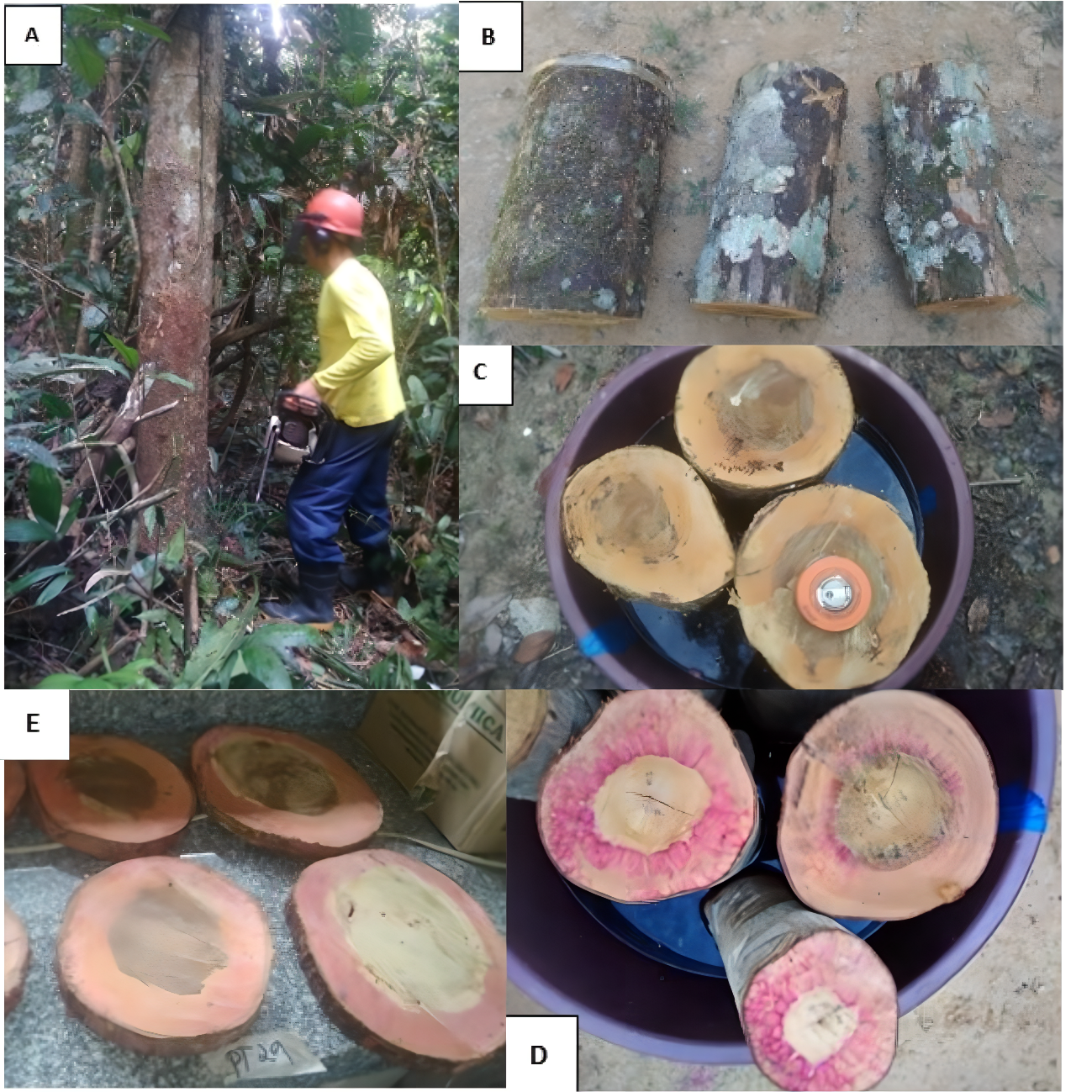


**Supplementary Figure 1**: Detailed flowchart of sapwood area measurement process. This figure depicts the sequential steps involved in quantifying the active xylem area, commonly referred to as sapwood area, including (A) tree selection and felling, (B) precise extraction of samples, (C) immersion in acid fuchsin dye for enhanced visibility, (D) monitoring dye ascent to delineate sapwood, (E) and retrieval of discs for conductive area analysis.


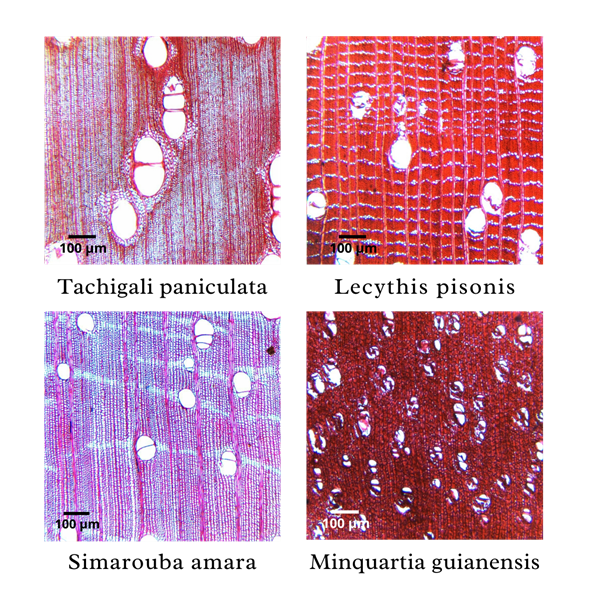


**Supplementary Figure 2.** Example images of stem xylem anatomy. *Simarouba amara* and *Tachigali paniculate* had the fastest growth rates and *Lecythis pisonis* and *Minquartia guianensis* had the slowest growth rates.
